# Electrolyte Solvation Structure Design for Sodium Ion Batteries

**DOI:** 10.1002/advs.202201207

**Published:** 2022-06-05

**Authors:** Zhengnan Tian, Yeguo Zou, Gang Liu, Yizhou Wang, Jian Yin, Jun Ming, Husam N. Alshareef

**Affiliations:** ^1^ Materials Science and Engineering Physical Science and Engineering Division King Abdullah University of Science and Technology (KAUST) Thuwal 23955‐6900 Saudi Arabia; ^2^ State Key Laboratory of Rare Earth Resource Utilization Changchun Institute of Applied Chemistry Chinese Academy of Sciences Changchun 130022 P. R. China

**Keywords:** electrolytes, sodium ion batteries, solvation structure

## Abstract

Sodium ion batteries (SIBs) are considered the most promising battery technology in the post‐lithium era due to the abundant sodium reserves. In the past two decades, exploring new electrolytes for SIBs has generally relied on the “solid electrolyte interphase (SEI)” theory to optimize the electrolyte components. However, many observed phenomena cannot be fully explained by the SEI theory. Therefore, electrolyte solvation structure and electrode–electrolyte interface behavior have recently received tremendous research interest to explain the improved performance. Considering there is currently no review paper focusing on the solvation structure of electrolytes in SIBs, a systematic survey on SIBs is provided, in which the specific solvation structure design guidelines and their consequent impact on the electrochemical performance are elucidated. The key driving force of solvation structure formation, and the recent advances in adjusting SIB solvation structures are discussed in detail. It is believed that this review can provide new insights into the electrolyte optimization strategies of high‐performance SIBs and even other emerging battery systems.

## Graphical abstract

This review focuses on the solvation structure of electrolytes in sodium ion batteries, elucidating the specific solvation structure design guidelines and their consequent impact on the electrochemical performance. The key driving force of solvation structure formation, and the recent advances in adjusting sodium ion battery solvation structures are discussed in detail.

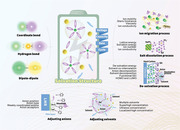

## 1 Introduction

Sodium ion batteries (SIBs) have become one of the most appealing and viable battery technologies due to the abundant and widely‐distributed sodium reserves compared to lithium used in lithium ion batteries (LIBs).^[^ ^1^, ^2^, ^3^, ^4^, ^5^ ^]^ Moreover, SIBs can use the widely available and lighter aluminum, rather than copper, current collector and hard carbon from renewable sources as anode, thus reducing the overall cost.^[^ ^6^, ^7^ ^]^ During the past decade, tremendous efforts have been exerted in the development of electrode materials for sodium ion storage, including new anodes (e.g., hard carbon, phosphides, metal oxides, and intermetallic materials)^[^ ^2^, ^8^, ^9^, ^10^, ^11^, ^12^ ^]^ and cathodes (e.g., sodium layered oxides, sodium layered phosphates, sodium layered sulfates).^[^ ^5^, ^13^, ^14^ ^]^ However, similar to LIBs, the electrolyte engineering in SIBs has received disproportionately small attention compared with electrode materials.^[^ ^15^, ^16^, ^17^, ^18^ ^]^ The reason why electrolytes in SIBs have not been extensively studied is the difficulty in establishing relationships between the molecular scale electrolyte kinetics with macroscopic battery performance. For instance, various electrolyte additives (e.g., FEC (fluoroethylene carbonate), TMP (trimethyl phosphate)) show great battery performance enhancement,^[^ ^19^, ^20^ ^]^ but the root causes for such enhancement remain inconclusive. Likewise, how the interactions between cations, anions, and solvent molecules improve rate and cycle performance is still not fully understood. Fortunately, the emergence of solid electrolyte interphase (SEI) theory afforded a feasible avenue to analyze the micro‐dynamics of electrolyte components.

**Figure 1:**
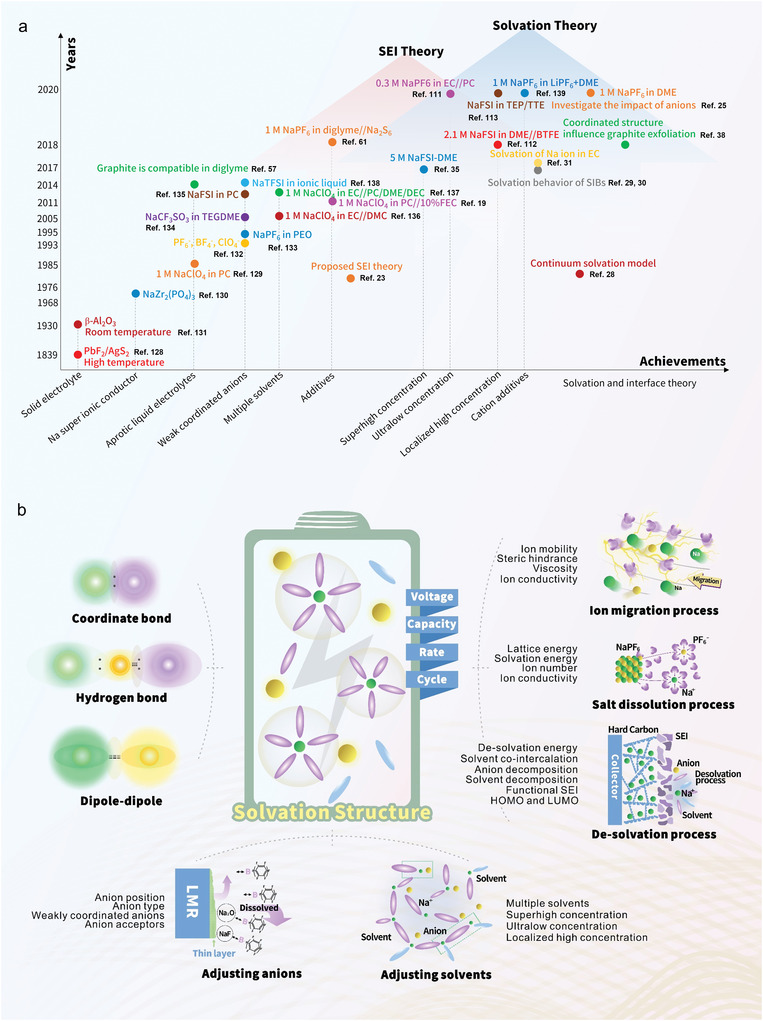
a) Key developments of sodium ion battery electrolytes and year in which the achievement was reported. b) Scheme showing the electrolyte solvation design issues for sodium ion batteries (SIBs) (Green: cations; yellow: anions; purple: solvents; blue: solvents).

The SEI concept was proposed in 1979 as a layer consisting of insoluble products of the reaction between metal anode and electrolyte solution. The SEI theory bridges battery macro‐performance and electrolyte micro‐behavior well.^[^ ^21^ ^]^ The physical and chemical properties of SEI (such as mechanical strength, porosity, HOMO level and LOMO level, etc.) can be successfully adjusted by introducing different functionalized electrolyte ingredients, in turn optimizing the voltage window, cyclic stability, and rate performance.^[^ ^22^, ^23^ ^]^ Nevertheless, very recently reports indicate that SEI theory alone sometimes cannot fully explain specific experimental phenomena; for instance, even after the formation of SEI, some graphite exfoliation,^[^ ^24^ ^]^ capacity decay of alloying anode (e.g., Sn,^[^ ^25^ ^]^ Sb^[^ ^26^ ^]^), or low Coulombic efficiency of metal plating/stripping^[^ ^27^ ^]^ can still be observed once the used electrolyte become incompatible. Therefore, an older concept was revived to explain the battery electrolyte behavior, including solvation and interface theories. The earliest research on solvation theory dates back to 1981, when Miertus et al. first proposed the continuum model, based on electrostatic interactions, to describe the solvation behavior in solution (**Figure** **1**a).^[^ ^28^ ^]^

However, for SIBs the earliest research on solvation theory in SIB electrolyte was conducted in 2015. Shakourian et al. investigated the Na^+^ ion solvation in alkyl‐carbonate electrolytes.^[^ ^29^, ^30^ ^]^ Subsequently, Pham et al. reported that Na^+^ ion generally exhibits a weaker interaction with the same solvents compared to Li^+^ ions, in turn inducing different dynamics in 2017.^[^ ^31^ ^]^ However, most reports focus on theoretically discussing the probable influence of solvation structure on SIB performance, with relatively few experimental verifications. Recently, “concentrated electrolytes” (employing the superhigh concentration electrolyte to modulate the interaction between cations and solvents) were used in SIBs, and successfully opened the door of optimizing battery performance using solvation theory combined with SEI theory.^[^ ^32^, ^33^, ^34^, ^35^ ^]^ In addition, in 2018, Ming et al. re‐examined the role of solvent molecules, anions, and additives in the electrolyte from the perspective of solvation theory, and provided several experimental demonstrations showing how solvation structure and interfacial behavior can be used to optimize battery performance.^[^ ^36^, ^37^, ^38^, ^39^ ^]^

Although, the electrolyte solvation design is very important and has aroused extensive research interest, there is no article that summarizes the solvation design principle and direction in the electrolyte engineering area for SIBs. Therefore, in this we provide the first summary of SIB behavior from the perspective of electrolyte solvation behavior. We first illustrated the driving force behind the solvation (coordination bond, dipole interactions, hydrogen bond), where the properties of the basic components of electrolyte (i.e., metal salts, solvents, additives) and their interactions (e.g., the coordination between Na^+^ ions and anions, the coordination between Na^+^ ions and solvents, the interaction between solvents and solvents) are analyzed in detail. Then, the solvation structure of Na^+^ ion is depicted, including the geometric parameters and thermodynamic descriptors. More importantly, the correlation between solvation structure and observed performance is explained. Finally, the strategies that can be used to adjust the solvation structure to optimize battery performance are summarized (Figure 1b). We believe this review presents a fresh perspective combining both theoretical and experimental approaches to leverage electrolyte solvation design to improve SIB and other mobile ion battery performance.

## 2 Requirements of Ideal Electrolytes

### 2.1 Sodium Salts

Metal salts, as the dominant constituents of the electrolyte, play a significant role in determining the electrochemical performance of SIBs. The role of metal salts in the electrolyte of SIBs includes the following aspects: 1) The metal salts serve as part of charge carriers being transported between the two separated electrodes. These carriers determine the electrolyte ionic conductivity; a poor ionic conductivity can degrade many battery parameters (e.g., it can dramatically increase the electrochemical polarization). 2) The sodium salts can affect the composition of the SEI, which compositions may be dissolved and destructed during cycling, thereby reducing the stability of SIBs. 3) The sodium ions arising from sodium salts participate in the intercalation (deintercalation) reactions within the electrode materials. Accordingly, the solvation structure of sodium ions, including the geometric morphology and delocalized electron density, could significantly impact diffusion of sodium ions, especially through the electrode/electrolyte interface. Likewise, in the bulk electrolyte, the chemical and thermal stability of electrolyte is influenced by the solvation structure of sodium ions to some extent.^[^ ^25^, ^40^ ^]^ 4) Aside sodium ion conductivity, SEI composition, and solvation structure of sodium ions, the thermodynamic HOMO energies of anions upon oxidation could limit the potential window of electrolytes to a certain extent, thus limiting the total energy density of SIBs. 5) The chemical toxicity and corrosive nature of most sodium salts has important ramifications for battery safety in practical applications.

In view of the considerations mentioned above, an ideal sodium salt should exhibit several characteristics. First, it is high solubility, which could achieve favorable ionic conductivity. The conductivity depends on two parameters, including number of free‐moving ions and their speed. As for the former, the total number of ions is determined by the solubility the sodium salt. For the latter, the properties of the solvent control the speed of moving cations and anions in salts (for example, dielectric constant and viscosity of solvents). Apart from dielectric constant of solvents, it is worth noting that the valence of cations and anions present in the electrolyte also influences the mobility to a certain degree. Nevertheless, owing to the monovalent nature of Na^+^ ion, the valence or oxidation state of the cations/anions is not important. Therefore, we focus on the solubility of the salts.

**Figure 2:**
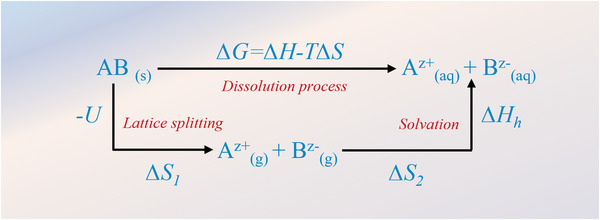
Energy parameters that are considered during the dissolution process. They include the salt lattice energy (*U*) and solvation energy (Δ*H*_h_).

Generally, the dissolution process of various sodium salts could be divided into two individual processes, encompassing the dissociation (splitting) of the lattice determined by lattice energy (*U*) and solvation with solvents determined by solvation energy (*∆H*_h_). The simplified Born–Haber cycle of dissolution process is shown in **Figure** **2**, where *∆H = −U + ∆H*_h_ and *∆S = ∆S*_1_ *+ ∆S*_2_. According to this cycle, the smaller Gibbs free energy (*∆G*) represents that the dissolution process is easier to proceed. In this regard, higher lattice energy decreases solubility of the salt, while higher solvation energy increases its solubility. For instance, in a typical aprotic solvent, sodium salts such as NaCl and NaF, are almost insoluble. The reason for this is the very large *U* resulting from the strong ionic bonds between the two atoms with great difference in electronegativity. On the contrary, the enhanced polarization of ionic crystals (reduction of the difference of electronegativity) will result in the transformation of ionic bonds to covalent bonds, which is beneficial for the dissolution of salts in aprotic solvents.^[^ ^41^ ^]^

Another widely accepted concept involves weakly coordinating anions (WCAs),^[^ ^42^, ^43^ ^]^ such as [CF_3_SO_3_]^–^, [BF_4_]^–^, [ClO_4_]^–^, [AlX_4_]^–^, [MCTFSI]^–^, or [MF_6_] (X = Cl–I; M = P, As, Sb, etc.). In such anions, the negative charges are delocalized over the anions, which allow us to regulate the electronegativity via utilizing electron withdrawing substituent to replace the atoms in anions. Therefore, the interaction of charge delocalized anions and cations will be significantly reduced, and the *U* will be reduced, thereby improving solubility.

The second desired characteristic of sodium salts is electrochemical stability. In theory, for maintaining the thermodynamic stability of an SIB, the electrochemical window (ESW) of electrolytes should extend beyond the redox potential of the anode and cathode. ESW is defined as the difference between the lowest unoccupied molecular orbital (LUMO) and the highest occupied molecular orbital (HOMO). In view of this, the variety of anions in sodium salts has a significant effect on the ESW of electrolyte. For instance, in a mixture solvent of EC/DEC, the oxidation potential follows the sequence NaPF_6_ > NaClO_4_ > NaTFSI > NaFTFSI > NaFSI,^[^ ^44^, ^45^ ^]^ which means that the PF_6_^–^ ion exhibits the lowest HOMO level (−11.67 eV) and is not easy to lose electrons and decompose. As for ClO_4_^–^ ion, the HOMO level locates at −7.89 eV, representing the poor chemical stability upon the oxidation process. However, the chemical stability is relative, which denotes the absolute stable anions that do not exist in an SIB. In addition, according to the previous reports, the anions of sodium salts can participate in SEI formation. For example, the decomposed product of PF_6_^–^ ion during the reduction process, NaF, is the main component of SEI.^[^ ^46^ ^]^ In brief, the anions in sodium salt can influence the chemical stability of electrolytes in two ways: one is that the HOMO level of anions limits the highest electrochemical window of SIBs; the other one is that the LUMO level of anions promotes the formation of SEI to preclude further electrolyte decomposition.

Another desired feature of the ideal sodium salt is the good thermal stability and low toxicity. For practical applications, where a large number of SIBs are expected to be deployed, safety is very important. Thus, sodium salts should have excellent thermal stability. Eshetu et al. investigated the thermal stability of sodium salts, which showed the following thermal stability trend: NaClO_4_ > NaBF_4_ > NaTFSI > NaPF_6_ > NaFTFSI > NaFSI.^[^ ^47^ ^]^ Obviously, NaClO_4_ exhibits the highest thermal stability. Nevertheless, NaClO_4_ is rarely utilized practically because of the strong oxidation properties (trend to give electrons) and explosive nature in the dry state. In addition, it is imperative to take the toxicity into account for the widespread implementation of SIBs. For instance, AsF_6_^–^ and SbF_6_^–^ based sodium salts are hardly used due to the toxic side products.

**Figure 3:**
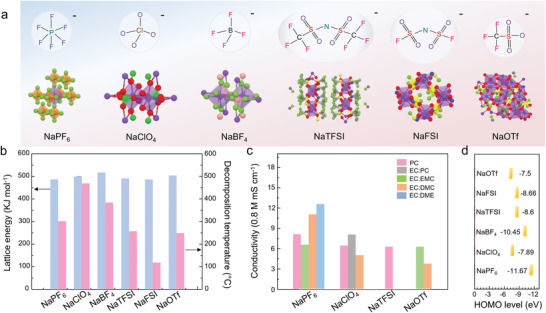
Chemical and physical properties of common sodium salts. a) Geometric configuration. The 3D structure is originated from Material Project database. b) Lattice energy and decomposition temperature. c) Ionic conductivity. d) HOMO level (Purple: Na; orange: P; red: O; pink: B; blue: N; grey: C; yellow: S; for NaPF_6_, NaBF_4_, NaTFSI, NaFSI, and NaOTf, the green color is F; for NaClO_4_, green is Cl) (NaTFSI, sodium bis(trifluoromethanesulfonate) imide; NaFSI, sodium bis(fluorosulfonyl)imide; NaOTf: sodium trifluoromethanesulfonate).

In short, the relative parameters of common sodium salts are summarized in **Figure** **3**. For the six metal salts, in general, the crystal with smaller lattice energy exhibits the highest conductivity (NaPF_6_ > NaClO_4_ > NaTFSI/NaOTf/> NaBF_4_). In addition, the HOMO level of the sodium slats follows the trend: NaOTf > NaClO_4_ > NaTFSI > NaBF_4_ > NaPF_6_, which shows that NaOTf and NaClO_4_ are more prone to be oxidized limiting the voltage window of SIBs. Therefore, the requirement of dissolution in organic solvent screens the majority of sodium salts. When taking the oxidation, reduction, thermal stability and toxicity into account together, the most suitable sodium salts can be narrowed down. Our analysis shows that NaPF_6_ offers the best compromise.^[^ ^48^ ^]^

### 2.2 Organic Solvents

Solvents provide an essential medium for the transport of ions arising from the dissociation of sodium salts. As same with LIBs, the low reduction potential of metal Na and high oxidation potential of cathode materials, precludes the use of protic solvents in the electrolyte. Therefore, aprotic solvents such as organic solvents, ionic liquid and solid ceramic electrolytes are considered more suitable candidates in SIBs. In this review, we focus on the most widely used liquid organic solvents. The organic solvents could affect the battery performance through the following factors: 1) As the medium of soluble sodium salts, the conductivity is greatly influenced by solvent chemistry. 2) The solvent could participate in the formation of functional SEI, which could impact coulombic efficiency and cycle durability. 3) More importantly, organic solvents determine the solvation structure of sodium ions, which then controls the de‐solvation behavior at the interface between the electrolytes and electrodes. 4) Likewise, the electrochemical stability of solvent molecules can limit the voltage window of SIB similar to sodium salts. 5) Since the organic solvent is the sole liquid component in an SIB, its instability (volatility, flammability) and toxicity can affect practical applications.

Therefore, the optimal organic solvent should have the following characteristics. i) High dielectric constant, low viscosity, and moderate Lewis's acidity/basicity, which could achieve favorable conductivity. According to the discussion in the section of sodium salts, the conductivity is determined by both of the number of free‐moving ions and the ion mobility. The former is decided by the solubility of sodium salts which is influenced by both *U* and *∆H*_h_. Unlike *U*, which is mainly affected by the inherent structure of the salts, *∆H*_h_ is mainly affected by the interaction between solvent molecules and solute ions. The dielectric constant, which is a macroscopically measured parameter defined as the capacity to separate the ion pairs in electrolyte, shows a positive correlation with *∆H*_h_. Thus, for salts with the same *U*, the solvents with higher dielectric constant result in better salt solubility, in turn achieving favorable conductivity. The Lewis acidity/basicity (electron acceptor/donor ability) of solvent molecules is another parameter that can influence *∆H*_h_. In theory, solvent molecules with strong Lewis's basicity should promote the solvation process of sodium salts. This is because of the possible coordination between the Na^+^ ion (with empty orbit) and solvent molecules with lone pair, thus increasing the solubility of salts. However, if the interaction between the Na^+^ ion and solvent molecules is too strong, it might result in a difficult de‐solvation process, in which could lead to the co‐intercalation of Na^+^ ion with solvent molecules. Apart from impacting solubility, the solvents also play a significant role in the ion mobility. The solvent viscosity, which incarnates the inherent attraction between molecules, has a pronounced effect on the ion mobility. According to the report of An et al.,^[^ ^49^ ^]^ the decrease of viscosity would produce as much as an order of magnitude increase in conductivity.

Another desired property of solvents for SIB is elevated electrochemical stability. Analogous to the anions in sodium salts, the oxidation and reduction of solvent molecules upon the charge/discharge process compete with the redox reactions of the electrode. The HOMO and LUMO of the solvents determine the stable ESW, that is, at electron energies higher than LUMO, the solvent is reduced and at electron energy levels lower than HOMO, the solvent is oxidized. More accurately speaking, we used the difference between oxidation and reduction potential of solvents to express the ESW according to the opinion of Peljo et al.^[^ ^50^ ^]^ In a word, the solvent potential difference is required to be large. However, a more general opinion suggests that the decomposition of solvents is not all notorious. The reduction of solvents near the anode could be beneficial for the formation of functional SEI. The reduced solvent molecules decompose into chemically active radicals that form new chemical entities coated on the surface of anode.^[^ ^51^, ^52^ ^]^

Other desired organic electrolyte properties include high safety with low melting point, high boiling point, high flash point, low toxicity, and low pollution impact. The solvent melting point is particularly important because the temperature of the battery can be extreme. The condensation of the solvents will cause the battery to fail when the surrounding temperature is lower than the melting point. For instance, EC solvent has a melting point of 36.4 °C, which is hardly used alone because it is solid at room temperature. The boiling point influences the volatility of electrolyte, in turn affecting the durability of an SIB due to the drouth of solvents. Moreover, the flash point is important due to the risk of spontaneous combustion of batteries. Finally, nontoxic and environment‐friendly solvents are essential for successful commercialization.

**Figure 4:**
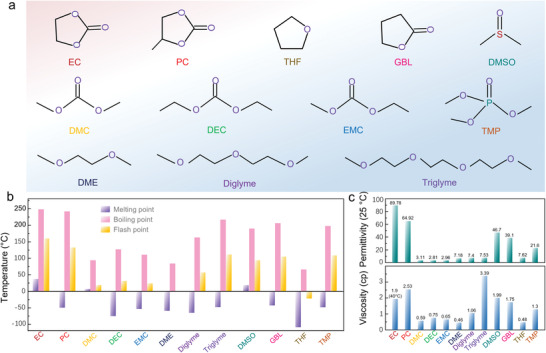
Structure and properties of organic solvents used in SIBs. a) Geometrical structure. b) Melting point, boiling point and flash point. c) Permittivity and viscosity (EC: ethylene carbonate; PC: propylene carbonate, DMC: dimethyl carbonate; DEC: diethyl carbonate; EMC: ethyl methyl carbonate; DME: dimethoxyethane; Diglyme: diethyleneglycol dimethylether; Triglyme: triethylene glycol dimethyl ether; DMSO: dimethyl sulfoxide; GBL: *γ*‐butyrolactone; THF: tetrahydrofuran; TMP: trimethyl phosphate).

**Figure** **4** summarizes the physical chemical parameters of several organic solvents used in SIBs, encompassing the common carbonate ester‐based solvents (such as EC, PC, DMC, DEC, EMC, and GBL) and ether‐based solvents (DME, diglyme, triglyme, and THF). Also, heteroatom‐based solvents such as sulfur coordinated solvent molecules (DMSO) and phosphorus coordinated solvent molecules (TMP) are listed. As shown in Figure 4c, EC as a typical carbonate ester‐based solvent, exhibits the highest dielectric constant indicating is strong dissolving ability for sodium salts. However, it is solid at room temperature, meaning elevated viscosity thus restraining the ion mobility. On the contrary, DEC and EMC display relatively low viscosity but low dielectric constants. Hence, reconciling two or three different solvents to optimize the electrochemical properties of electrolytes generally is a widely implemented strategy. For instance, Ponrouch et al. added low‐viscosity DMC into binary EC/PC solvent synthesizing the ternary EC_0.45_/PC_0.45_/DMC_0.1_ solvent, which significantly improved the ionic conductivity from 6.2 to 10 mS cm^−1^.^[^ ^15^ ^]^ According to the investigation of Bommier et al., the most discussed electrolyte solvents in published papers are EC/DEC, EC/PC, PC, EC/DMC.^[^ ^53^ ^]^ Nevertheless, the most frequently utilized solvents mentioned above seems do not represent the optimal solvent candidate.

One great advantage for choosing solvent in SIBs is that there is no Na^+^‐solvent co‐insertion occurred in carbonate ester‐based solvent, where the graphite‐like layer exfoliation can be inhibited even in PC‐based electrolyte.^[^ ^54^ ^]^ Likewise, the Na^+^ cannot be (de‐)intercalated within graphite anode due to the slow kinetics neither.^[^ ^55^, ^56^ ^]^ This is the reason why the hard carbon was used in SIBs, rather than the graphite anode. The first attempt to use ether‐based solvents in SIBs by Jache et al. found that the graphite is compatible in diglyme based electrolyte, which was credited to the co‐intercalation of Na^+^ ion and diglyme molecules forming thermodynamically stable ternary Na(diglyme)_2_C_20_ product.^[^ ^57^ ^]^ Furthermore, glyme combinations such as triglyme, tetraglyme, di(propylene glycol)methyl ether, diethylene glycol dibutyl ether (Butyl‐2G), and 1,5‐dimethoxypentane (1,5‐DMP) were developed recent years.^[^ ^54^, ^58^ ^]^ Apart from the ester‐based and ether‐based solvents, the phosphorus‐based TMP solvent exhibits a broadened liquid temperature range, which frequently is applied as nonflammable electrolyte in SIBs.^[^ ^20^ ^]^

In summary, developing optimal solvents is a crucial objective for achieving an SIB with favorable electrochemical performance. Choosing the optimal solvents requires comprehensive consideration encompassing slat dissolution, solvation/de‐solvation characteristics, compatibility with electrode materials, practical working environment such as the extremely low temperature or high temperature and working voltage window.

### 2.3 Additives

Analogous to LIBs, electrolyte additives can be used to tune the electrochemical performance of SIBs. These additives can affect SIB in various ways: 1) Significant impact on the electrode/electrolyte interface. According to the commonly accepted opinion, the additives participate in the SEI formation which influences the electrochemical performance.^[^ ^19^, ^59^, ^60^, ^61^, ^62^ ^]^ Recently, in stark contrast to the SEI effect, Ming et al. proposed an alternative innovative view in which additives are able to change the de‐solvation process of cations near the interface (see more detail in the next section).^[^ ^38^, ^60^, ^63^ ^]^ 2) The additives can change the solvation structure of Na^+^ ions, which can change ionic conductivity of the electrolyte, electrochemical stability of solvents and sodium salts, and viscosity.^[^ ^59^, ^64^ ^]^ 3) Functional additives are aimed at mitigating some specific drawbacks of primary electrolytes, such as resisting overcharging,^[^ ^65^ ^]^ suppressing flammability,^[^ ^66^, ^67^ ^]^ and maintaining working at extremely low temperature.^[^ ^62^ ^]^

Hence, introducing additives into the electrolyte should take the following considerations into. i) Small amount. Generally, the weight ratio of additives should be maintained below 5% because a higher ratio would influence the original electrolyte composition, meaning the additive could dominate the electrochemical behavior.^[^ ^68^ ^]^ ii) The additive should facilitate the forming of a durable SEI. In other words, the decomposed products of additives at low potential should participate in SEI formation to reduce the irreversible capacity and side reactions. iii) Specific function additives have unique requirements. For example, anti‐overcharge additives require the additive molecules to be oxidized reversibly at slightly higher potentials than the normal end‐of‐charge potential of the positive electrode; flame retardant additives require the additive molecules is capable to terminate radical chain reactions responsible for the combustion reaction in the gas phase and viscosity diluter additives, etc.

**Table** **1** summarizes heterogeneous electrolyte additives which have been applied in SIBs. Surprisingly, the fluoroethylene carbonate (FEC) additive is the most widely‐used among the various functional additives since its first successful investigation by Komaba et al. in 2011.^[^ ^19^ ^]^ The main reason for this success is the stable SEI formation on the surface of hard carbon due to the decomposition of FEC. However, in stark contrast to LIBs, the difluoroetyhene carbonate (DFEC), ethylene sulfite (ES), and vinylene carbonate (VC) additives (already widely applied in LIBs) have a detrimental impact on SIB performance. Nevertheless, Zhang et al. found that VC is effective in inhibiting interface polarization in SIB, thus forming a more robust SEI on the MoO_2_ anode surface.^[^ ^60^ ^]^ Besides additives added to promote more stable SEI, other additives target the cathode–electrolyte interface (CEI). For example, adiponitrile (APN) has been used because it has a stronger electron donating ability compared with carbonate solvents, thus it is easier to be oxidized on the surface of the cathode material to form stable CEI.^[^ ^62^ ^]^ Moreover, additives with flame retardant ability, conductivity enhancement, scavenger, and overcharge are listed in Table 1. It is worthy to note that reports on electrolyte additives in SIBs are sporadic compared with the extensive literature on LIBs additives.

**Table 1:** Electrolyte additives and their role in SIBs

Function	Additive	Beneficiary
Promoting SEI formation	FEC (fluoroethylene carbonate)^[^ ^19^ ^]^ PST (prop‐1‐ene‐1,3‐sulton)^[^ ^59^ ^]^ DTD (1,3,2‐Dioxathiolane‐2,2‐dioxide)^[^ ^59^ ^]^ VC (vinylene carbonate)^[^ ^60^ ^]^ TMSP (tris(trimethylsilyl) phosphite)^[^ ^69^ ^]^ Rubidium and cesium salts^[^ ^70^ ^]^ water (in ionic liquid electrolyte)^[^ ^71^ ^]^ NaNO_3_^[^ ^61^ ^]^ SbF_3_^[^ ^72^ ^]^	Hard carbon and alloy anode Hard carbon anode Hard carbon anode MoO_2_ anode Sn_4_P_3_ anode Hard carbon anode Na metal anode Na metal anode Na metal anode
Promoting CEI formation	AND (adiponitrile)^[^ ^62^ ^]^ NaNO_2_^[^ ^73^ ^]^	Na_0.76_Ni_0.3_Fe_0.4_Mn_0.3_O_2_ Na_0_[Co_0.05_Mn_0.95_]O_2_
Flame retardant	F‐EPE (2,2,3,3‐tetrafluoropropyl ether), EFPN (ethoxy(pentafluoro)cyclotriphosphazene)^[^ ^67^ ^]^	Na_0.44_MnO_2_ cathode and Na metal anode
Conductivity enhance	EMImFSI (1‐ethyl‐3‐methylimidazolium bis(fluoromethanesulfonyl)imide)^[^ ^74^ ^]^	Hard carbon anode
Scavenger	H‐ZSM‐5 zeolite^[^ ^64^ ^]^ TMSPi (tris (trimethylsilyl) phosphite)^[^ ^75^ ^]^	Na_a_MO_2_ cathode and hard carbon anode Na_3_V_2_(PO_4_)_2_F_3_ cathode and hard carbon anode
Overcharge protection	Biphenyl^[^ ^65^ ^]^	Na_0.44_MnO_2_ cathode and Na metal anode

In the previous sections, we have discussed sodium salts, organic solvents, and additives in SIBs. The ideal design requirements of these electrolyte constituents must take several factors into account. In the next section we discuss interaction of these electrolyte components and solvation structure formation.

## 3 Molecular Interaction and Solvation

### 3.1 Typical Interactions between Electrolyte Components

#### 3.1.1 Coordinate Bond

**Figure 5:**
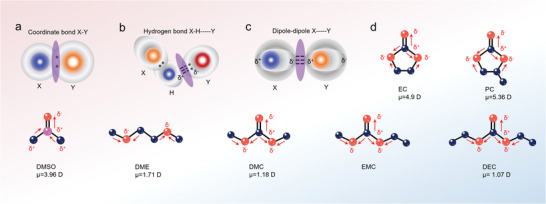
Schematic depicting interaction and bonding present in the electrolyte. a) Coordinate bond. b) Hydrogen bond. c) Dipole to dipole interaction (the gray shadow represents the electron cloud of atoms). d) Dipolar moments (*μ*) of the typical solvent molecules (the arrow points from the center of positive charge to the center of negative charge).

Short‐range interaction is considered to be an intensive force between the sodium ion and the center of solvent molecules, dramatically decreasing with distance. In general, coordinate bond, as a typical short‐range force, is taken into account, which describes a dative covalent bond where both of the electrons arise from the same atom (Seeing in **Figure** **5**a). By the electrostatic attraction of nuclei and electron pairs, the coordinate bond shows high strength similar with covalent bond. It is possible to exist between the salts and solvents in the electrolyte. Cations such as Na^+^ ion have empty 2s orbitals after losing the outermost electrons, while some of the organic solvent molecules are extreme electron donors being able to donate lone electron pair (e.g., adiponitrile). On the basis of the electron sharing of coordinate bond, the capability to donate lone electron pair of solvents determines the strength of coordinate bond. In other words, the Lewis base of organic solvents determines the strength of the coordinate bond where a higher Lewis base leads to tighter interaction.

In addition, the coordinate bond is commonly present between anions and cations in sodium salts based on various WCAs. The delocalization of electrons on the anions by the presence of electron withdrawing groups induces significant weakening of the coordinate bond strength between the Na^+^ ion and anions. In this regard, the coordinate bond formation capability can be altered by choosing different electron withdrawing groups to change the donor number of anions, in turn altering the degree of electron delocalization.

#### 3.1.2 Hydrogen Bond

A hydrogen bond also is a short‐range interaction but much weaker than the coordinate bond. It describes an electrostatic attraction between a hydrogen atom, which is covalently bound to a more electronegative atom or group (X), and another electronegative atom (Y) bearing a lone pair of electrons. As shown in Figure 5b, the hydrogen bond originates from the dipole orientation on H atoms due to the strong electron attraction ability of X atoms, thus resulting in the electrostatic force between H atoms and Y atoms. The formation of hydrogen bond between anions from sodium salts and solvent molecules was proposed by Schroder et al.^[^ ^76^ ^]^ Particularly, for fluorinated ions or species with at least one lone pair (tributylamine, bis(oxalato)borate), the strong interaction with solvent hydrogen atoms (e.g., the hydrogen atoms of the propylene group in PC) is observed. Besides, the hydrogen bonding could be established between the organic solvent molecules and additive molecules, thereby inducing the distinguishing solvated structure to influence the electrochemical performance of SIBs.

#### 3.1.3 Van der Waals Forces

The van der Waals forces (denoted as electrostatic interaction sometimes) are fairly ubiquitous among sundry electrolytes arising from the electrostatic attraction or repulsion between the adjacent positive and negative charge centers. Therefore, these bonds are weaker than the coordinate bond. The typical van der Waals forces can be divided into three different interactions: i) Dipole–dipole force, which arises from the permanent dipoles of the two adjacent molecules or atoms. It mostly stems from the balance of electrostatic attraction and repulsion forces, usually produced in two polar molecules. For the aprotic solvents used in the electrolytes of SIBs, the dipole–dipole force is related to the dielectric constant of solvents. As shown in Figure 5, the solvents are considered to be nonpolar for 0 < *ε* < 5, such as DMC, DEC, and EMC. The solvents are considered to be medium polar when 5 < *ε* < 30, such as DME, Diglyme, and Triglyme. The solvents are considered to be polar when *ε* > 30, such as EC, PC, and DMSO. Due to the strong dipole effect, the dipole–dipole forces dominate the van der Waals interactions in the polar solvents.^[^ ^77^ ^]^ ii) Dipole‐induced force is similar to the dipole–dipole force but one of the dipoles is induced by the near permanent dipole owing to the deformation of the electron cloud by the attraction of positive charge center. This bond type is widely observed between polar molecules as well as between polar molecules and non‐polar molecules. iii) Dispersion force, is a universal interaction among all the atoms and molecules, originating from the instantaneous dipole formation attributed to the uneven electron distribution at a given moment.

It is worth pointing out that the three different van der Waals forces could be influenced by different factors. For instance, dipole–dipole force mainly is disturbed by the dipole moment which is determined by bond and electron density. In addition, dipole‐induced dipole force is dominated by both dipole moment of polar molecules and deformation of nonpolar molecules. As for the dispersion force, it is considered to be related to ionization energy and molecular deformability. Considering that the three interactions frequently occur at the same time in the electrolyte, typical analysis of the van der Waals forces do not distinguish between them.

A sketch of the dipolar moments (*µ*) present in common solvents (such as cyclic ester: EC and PC; linear ester: DMC, EMC, and DEC; ethers: DME) of SIBs is shown in Figure 5d. Typically, in EC and PC, the strong dipolar moments occur on the carboxyl oxygen atoms, arising from the high electronegativity of oxygen atoms compared with carbon atoms. The electron deflection near the double bond engenders a net negative charge (*δ* ^–^) on oxygen atom and a net positive charge (*δ* ^+^). Likewise, the ether oxygen atoms also exhibit a net negative charge due to the dipole effect, but the interaction is weaker due to the decreased sharing of electron cloud density. This effect could be validated by the DME molecules, which exhibit a lower dipolar moment of 1.71 D. It is worth noting the carboxyl carbon atom shows a relatively high electro‐positivity in this case, which makes them prone to interacting with the anions or the molecules with lone pair electrons invoking the hydrogen bond effect. Compared with EC and PC, linear esters DMC and DEC display a weaker dipolar moment (1.18 D and 1.07 D, respectively). This weak dipolar moment is likely due to the highly symmetric steric configuration. The dipolar moments correlate well with the statistic permittivity as shown in Figure 5. The higher dipolar moments result in higher permittivity indicating favorable interaction between the molecules.

Due to the existence of dipolar moments originating from the van der Waals' force, the interactions between the cations and anions, cations and solvent molecules, as well as solvent molecules and solvent molecules, are considered to be an electrostatic interaction. As an example, the electrostatic force between oxygen atom as the net negative charge center and Na^+^ ion as the positive charge center is the dominated drive force of solvated process. The classic physical model gives the typical potential energy of the three electrostatic force^[^ ^78^, ^79^ ^]^

(1) $$U_{\mathrm{ion}-\mathrm{ion}}=\frac{-(z_{1}z_{2}e^{2})}{4\pi \varepsilon_{0}r}$$

(2) $$U_{\mathrm{ion}-\mathrm{dipole}}=-\frac{ze\mu \cos\theta }{4\pi \varepsilon_{0}r^{2}}$$

(3) $$U_{\mathrm{dipole}-\mathrm{dipole}}=-\frac{2{\mu_{1}}^{2}{\mu_{2}}^{2}}{{\left(4\pi \varepsilon_{0}\right)}^{2}3Tr^{6}k_{B}}$$

where *U* is the electrostatic potential energy, *ε*_0_ is the dielectric constant, *z*_e_ is the charge of the ion, and *r* is the distance between positive charge center and negative charge center. The *μ* is the dipole moment, where *θ* is the dipole angle relative to the line joining the ion and the center of the dipole, *k*_B_ is the Boltzmann constant, and *T* is the absolute temperature. Obviously, the electrostatic interaction depends on the dipolar moment of molecules and the bond length, bond angle. This interaction can be modulated by two approaches. One approach is strengthening or weakening the dipole effect of the molecules. For instance, replacing the carbon atoms with the more electronegative fluorine atoms, would cause a decrease in electron density on the adjoining oxygen atoms, thereby impairing the electronegative charge center. A second approach to modulate the electrostatic interactions is the distance between the solvent molecules and Na^+^ ions. The size of solvent molecules impacts the steric resistance directly, which in turn causes perturbation to the van der Waals bond length.

### 3.2 Solvation Structure

#### 3.2.1 Solvation Shell

**Figure 6:**
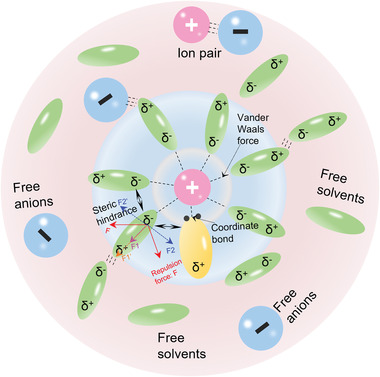
A model showing the solvation structure of the cations in SIB electrolyte.

Taking the typical interactions such as coordinate bond and van der Waals force into account, a possible solvation structure model is shown in **Figure** **6**. Aiming at simplifying the model, an isolated cation is located at the center. In this case, the interactions between the cations and anions are neglected. The model is drawn as a sphere as we regard solvent as a continuous and uniform medium wrapping the solute ion. There are two solvation shells. The first solvation shell (shown by the inner circle) forms due to the strong electrostatic forces arising from the dipole (solvent molecules A) with the cation. Some solvent molecules with extensive Lewis's alkalinity (discussed in the prior section) also exhibit a strong coordinate interaction with the cations. Owing to the synergy between electrostatic forces and coordinate bonds, the attraction and repulsion reach an equilibrium thus forming the first solvation shell. It is worth pointing out that the “coordinated solvation shell” mentioned in some literature is based on the same concept as the first solvation shell. The forceful interaction and compact connection between the cations and solvent molecules cause the cation to move within the first solvation shell instead of migrating by itself.

Compared with the first solvation shell, the secondary solvation shell is less compact; it includes the partially restrained solvent molecules, the attracted anions, and even the ion pair. Likewise, the free solvent molecules and free anions are also contained in the secondary solvation shell. Notably, the proposed solvation structure is based on the assumption that the ion pairs can be separated by the solvents. Nevertheless, according to Griffiths and Wijayanayake,^[^ ^80^ ^]^ the permittivity of solvents has a remarkable influence on the ion pairs and the free ions or molecules. Typically, if the permittivity is less than 5, only the contact ion pairs are expected to be present. However, if the permittivity is larger than 23, the free ions and solvation shells are present.^[^ ^80^ ^]^ Both of the ion pairs and solvation shells exist for solvent permittivity between 5 and 23. Moreover, aside from considering the capability of the solvents, the extremely compact interactions between the cations and anions, such as the coordinated bonds, would sharply increase the amounts of ion pairs, thereby reducing the amounts of free ions. In short, the solvation structure is complicated encompassing electrostatic attraction (repulsion), coordination, polarization, and dispersion processes. The actual battery behavior observed in practice is the sum of all these interactions, where building a model is necessary to help us understand their behaviors and interactions.

#### 3.2.2 Geometrical Parameters

**Figure 7:**
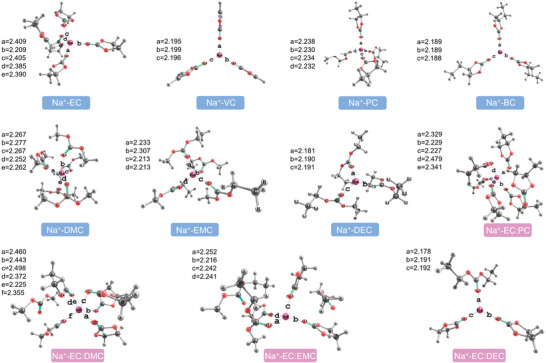
Geometries of the first solvation shell of Na^+^ ion in different solvents (including single solvents and mixture solvents); *a*, *b*, *c*, *d*, *e*, and *f* represent different bond lengths. Reproduced with permission.^[30^ ^]^ Copyright 2015, American Chemical Society.

Two key parameters are necessary to describe the geometrical arrangement of solvent molecules. One is the coordination number, which represents number of solvents molecules surrounding the cation in the first solvation shell. The second is the average bond length, which measured the distance between the centered sodium atom and the oxygen atom of solvents. Fard et al. proposed the solvated geometries of Na^+^ ion in different solvents based on the optimized theory of M06‐2X/6‐311++G(d,p) level (shown in **Figure** **7**).^[^ ^30^ ^]^ The primary solvation spheres exhibit distinct characteristics with different solvent molecules. For instance, in EC solvents, five solvent molecules surround each Na^+^ ion; in comparison, three VC solvents molecules surround each Na^+^ ion due to the larger size of VC molecules. In addition, the average bond length of Na^+^—O decreases from 2.359 Å (EC) to 2.195 Å (PC) due to the decreased steric hindrance between the solvent molecules. The steric hindrance can be understood as the repulsion force between adjacent solvent molecules in the first solvation shell due to the dipoles pointing in the same direction. As shown in Figure 6, the steric hindrance (F, red arrow) can be divided into two forces, one is along the direction tangent to the circle (F2, blue arrow), and the other along the centrifugal direction (F1, purple and orange arrows). Obviously, the F2 is counteracted by the adjacent solvents. However, the F1 forces are superimposed (they add up), which keeps the solvents away from the cations. Therefore, the large solvent size or high coordination number in the first solvation shell would result in large steric hindrance, thereby increasing the bond length between Na^+^ ions and solvents. Moreover, the simulations shed light on that with the addition of EC into PC, two PC and three EC molecules would stay in the first solvation shell. For EC/DMC mixture, two DMC and four EC molecules occupy the first solvation shell. Likewise, both the EC/EMC and EC/DEC solvents show similar behavior, which indicates that EC exhibits a more compact interaction with Na^+^ ions compared with the DEC, DMC, and EMC (which usually have a fairly weak dipole). The average bond length (distance between the centered sodium atom and the oxygen atom of solvents) is also changed upon addition of EC. The bond length in EC:PC solvent mixture increased from 2.233 to 2.321 Å due to the increased steric hindrance. On the contrary, the EC/DEC, EC/DMC, and EC/EMC show decreased bond length due to the decreased steric hindrance arising from the replacement of large sized DEC, DMC, and EMC by smaller sized EC molecules.

Apart from the impact of solvent molecules, the centered cation itself can exhibit profound effect on the geometrical solvation structure. Pham et al. analyzed the first solvation shell of different cations (Li^+^ ion, Na^+^ ion, and K^+^ ion) in EC solvent.^[^ ^31^ ^]^ The simulation displayed that the Li^+^ ion exhibits a well‐defined first solvation shell while the larger Na^+^ ion and K^+^ ion show more disordered and flexible solvation structures. These results illustrate that both the coordination number and average bond length increase with increasing cations size. According to Equation (1), the interaction between cations and solvent molecules is mainly electrostatic. Thus, the larger radius of cations should result in weaker force between the cations and the oxygen atoms of the solvents, which in turn induces disordered and flexible structure (including the increased coordination number and bond length).

#### 3.2.3 Thermodynamic Descriptors

Another approach to describe the solvation behavior in SIB electrolyte uses thermodynamic variables. These variables include:

1) Binding energy (Δ*E*_b_), which reflects the strength of the interactions between cations and solvent molecules. It could be calculated based on the difference in energy between the solvation complex and the constituents that make the complex (solvent molecule (SM) and Na^+^ ion):
(4) $$\Delta E_{b}=E_{\left(\mathrm{complex}\right)}-\left(n\cdot E_{\left(\mathrm{SM}\right)}+E_{\left(\mathrm{Na}+\mathrm{ion}\right)}\right)$$
2) Free energy of solvation (Δ*G*_sol_), which refers to the part of the reduced internal energy that can be converted into external work in a certain thermodynamic process. The solvation process is easier to occur for Δ*G*_sol_ < 0.

For the solvation process, Δ*G*_sol_ could be calculated using the following equation:

(5) $$\;\Delta G_{\left(\mathrm{sol}\right)}=G_{\left(\mathrm{complex}\right)}-\left(n\cdot G_{\left(\mathrm{SM}\right)}+E_{\left(\mathrm{Na}+\mathrm{ion}\right)}\right)$$

3) LUMO and HOMO energy levels (LUMO/HOMO), which denote the highest occupied molecular orbital (HOMO) and the lowest unoccupied molecular orbital (LUMO) of the solvated Na^+^ ion‐solvents. The energy difference between the HOMO and LOMO is defined as band gap.

These thermodynamic descriptors are summarized for sodium ions in different carbonate solvents in **Table** **2**, which is based on the simulations done by Fard et al.^[^ ^30^ ^]^ Obviously, the binding energy between the Na^+^ ions and EC molecules is largest (−115.73 kcal mol^−1^) among all single‐component solvents, denoting the strong interactions during the solvation process. In contrast, DEC exhibits the smallest binding energy of −77.02 kcal mol^−1^, indicating a weak solvation process. The simulation results are consistent with our previous analysis that solvation interactions show explicit dependency on the dipole moments of solvent molecules. In addition, the Δ*G*_sol_ also illustrates a similar result where the solvation process is easier to occur for Na^+^ ion in EC solvents (−71.63 kcal mol^−1^). Fard et al. further clarified that Δ*G*_sol_ is proportional to permittivity in most solvents. This means that improving the permittivity of the solvents would benefit the solvation process of Na^+^ ions. Interestingly, the VC molecules do not fit such correlation due to their extremely large permittivity.

**Table 2:** Thermodynamic descriptors of different Na+ ion‐solvent complexes

Solvents	Δ*E*_b_ [kcal mol^−1^]	Δ*G*_sol_ [kcal mol^−1^]	LUMO/HOMO (pure solvents) [eV]
Na‐EC	−115.73	−71.63	−2.44/−12.7 (−0.38/−10.45)
Na‐VC	−79.22	−56.60	−2.83/−11.7 (−0.24/−8.82)
Na‐PC	−101.16	−69.36	−2.31/−12.77 (−0.37/−10.34)
Na‐BC	−88.08	−64.13	−2.52/−12.97 (−0.36/−10.29)
Na‐DMC	−97.71	−45.59	−2.76/−13.36 (−0.04/−10.3)
Na‐EMC	−88.68	−50.37	−2.87/−13.19 (−0.06/−10.22)
Na‐DEC	−74.08	−46.07	−1.84/−15.69 (−0.07/−10.14)

Moreover, on the basis of orbital energy and density of state (DOS) calculations, the HOMO and LUMO energy levels of several solvents (after solvation) are given in Table 2. It is worth noting that all Na^+^‐solvent complexes exhibit a negative shift (i.e., the lower HOMO compared to the isolated or bare solvent) after forming the solvation structure. This shift indicates that the solvent molecules become more resistant to oxidation after solvation with the Na^+^ ions. Also, we see that the Na^+^‐DEC complex shows a good thermodynamic stability with a large band gap of 13.85 eV, while the Na^+^‐VC shows inferior thermodynamic stability with a small band gap of 8.87 eV.

Aiming at investigating the solvation process, Okoshi et al. utilized the different simulation method (B3LYP/cc‐pVDZ(‐PP)) to calculate the solvation energy of Na^+^ ion (*∆E*_sol_) in various organic solvents.^[^ ^81^ ^]^ In fact, the solvation energy is equal in magnitude but opposite in sign to the binding energy, as mentioned before. The results indicate that the *∆E*_sol_ of Na^+^ ion is: APN (186.3 kJ mol^−1^) > DMSO (169.1 kJ mol^−1^) > PC (157.3 kJ mol^−1^) > EC (151.9 kJ mol^−1^) >DEC (147.5 kJ mol^−1^) > ATN (acetonitrile, 137.4 kJ mol^−1^) > NM (nitro methane, 118.1 kJ mol^−1^). Obviously, the common carbonates demonstrate a moderate solvation energy which is considered to be the favorable solvent candidates for both solvation and de‐solvation process. By fitting and analysis, Okoshi et al. further concluded that there is a linear relationship between the chemical hardness (*η*), electrostatic potential (*σ*) and the *∆E*_sol_.

(6) $$\Delta E_{\mathrm{sol}}=-24.7\eta +286.6$$

(7) $$\Delta E_{\mathrm{sol}}=0.37\sigma +114.3$$

These relationships indicate that increasing the electrostatic potential or decreasing the chemical hardness of solvent molecules confers a feasible avenue to improving the solvation energy.

## 4 Impact of Solvation Process on the Electrochemical Performance

**Figure 8:**
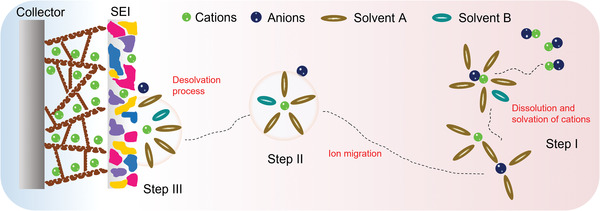
Illustration of the impact of solvation process on the electrochemical performance.

Based on the previous discussion, the solvation process can be divided into three separate steps (**Figure** **8**). These include the dissolution and solvation process of sodium ions in organic solvents (step I); this step has a clear impact on the electrolyte conductivity, as discussed in the section titled “sodium salts.” The next migration process of sodium ions in organic solvents occurs (step II); this step is also critically important for determining electrolyte conductivity. The third step is the de‐solvation process of sodium ions at the interface between electrolyte and electrode (step III); this process greatly influences the ion intercalation behavior. It is worth pointing out that all three steps are driven by the interaction between cations, solvent molecules, and anions. For instance, in step I, the interaction of anions and cations in salts decides the lattice energy of salt and the interaction of cations and solvent molecules decides the solvated energy. In this regard, on the one hand, the week coordinated anion‐based sodium salts are developed to enhance the solubility. On the other hand, the solvents with high donor number are investigated to improve the solubility of salts.

In step II, the mobility of sodium ion also is subject to the interaction between cations and anions as well as solvents and solvents. The strong coordination between the cations and anions would inhibit the transport of solvated sodium ions. Likewise, the intense interactions, such as hydrogen bond, between solvent molecules would result in the elevated viscosity, in turn suppressing the ion mobility. However, both the step I and step II only influence the electrolyte conductivity, thereby inducing the rate performance of SIBs decrease. On the contrary, the step III is a more intricate process, which encompasses the de‐solvated process, the decomposition of solvents and anions on the surface, striding across the energy barrier and passing through the SEI. These processes become the rate determining step, significantly affecting the capacity, rate performance, voltage window, coulombic efficiency, and cycle stability.

From the above discussion, it is clear that the solvation structure plays a crucial role in the electrochemical behavior of batteries. In the next section, several cases of modulating the solvation structure to optimize sodium ion battery performance are discussed.

## 5 Modulating the Solvation Structure to Optimize Battery Performance

The solvation structure of electrolytes essentially consists of cations, solvent molecules, and anions, as has been discussed in detail in Section 3. Therefore, the solvation shell could be modulated by adjusting these three ingredients to change the interactions between the different components, in turn having an impact on the step I, II, and III of electrochemical processes. Before analyzing various modulating strategies in detail, it is necessary to emphasize the difference between SEI theory and solvation theory.

### 5.1 SEI and Solvation Structure

**Figure 9:**
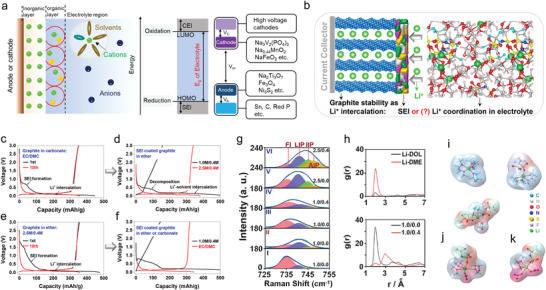
SEI theory and solvation theory. a) Illustration of SEI in a battery. b) Schematic view of the two proposed models to explain the stability of a graphite anode in an Li ion battery. c–f) SEI mediated electrochemical performance. g) Raman spectra of S‐N‐S bending motions for TFSI^−^ in an electrolyte using I) DME, II) DOL, and III–VI) DOL/DME as solvents. h) Top: RDF of Li^+^ to the oxygen of DME and DOL; Bottom: RDF of Li^+^ to the oxygen of TFSI^−^ in the electrolytes with (red) and without (black) NO_3_. i,j,k) Schematic view of the first shell of Li^+^ in different solvents, where DME is the dominant solvent component. Reprinted with permission.^[24^ ^]^ Copyright 2018, American Chemical Society.

As mentioned above, SEI is defined as the solid electrolyte interface, which was first proposed by Peled in 1979.^[^ ^21^ ^]^ Generally, SEI is composed of insoluble products of the reaction of metal anode with the electrolyte solution (**Figure** **9**a). This reaction is a thermodynamically spontaneous process, meaning that once the metal is immersed in the electrolyte, the metal surface will be covered with the SEI layer. The thickness of the layer is determined by the electron tunneling range. The SEI is typically electronically insulating but ionically conducting. Thus, once the initial SEI is formed, these characteristics suppress further decomposition of solvents and salts at the interface and extend the LUMO and HOMO levels as shown in Figure 9a. Subsequently, Smith. et al. revealed the dynamics of SEI formation process.^[^ ^82^ ^]^ They pointed out that the SEI is not constant, but rather the solvents and salts undergoes decomposition during the charge and discharge process to continuously replenish SEI. Since the SEI layer is not completely electronically insulated, electron‐tunneling or transfer can still occur across these interphases under certain conditions, such as reaching a sufficient polarization potential, as evidenced by the use of redox shuttle chemicals for the purpose of over‐charge protection. In addition, a more compact SEI structure can suppress the decomposition of the electrolyte, and the SEI is a natural barrier to prevent the co‐intercalation of solvent molecules and cations, thereby avoiding the incompatibility of electrolyte and electrodes. The SEI theory has been widely accepted in the metal ion batteries, such as LIBs, SIBs and PIBs.^[^ ^3^, ^18^ ^]^

Recently, some findings show that SEI can be influenced by the electrolyte solvation structure. The SEI derived from different solvation structure exhibits significant variation in its composition, film thickness, and morphology.^[^ ^83^, ^84^, ^85^, ^86^ ^]^ For instance, the cation solvation structure in ultrahigh concentration electrolytes will lead to the formation of an anion‐derived SEI.^[^ ^34^ ^]^ Such anion‐derived SEI shows great advantages compared with the traditional solvent‐derived SEI, such as stability and sodiation−desodiation kinetics.^[^ ^35^ ^]^ However, the influence of electrolyte solvation structure may not entirely control SEI properties. It is just another viewpoint the complements, not replaces, the traditional SE theory.

Hence, Ming et al. proposed a new concept to explain the graphite anode stability in LIBs.^[^ ^24^ ^]^ They argued that Li coordination in the electrolyte can explain the graphite anode stability instead of the conventional SEI theory (Figure 9b). Their experimental results showed that the graphite anode could be cycled stably in carbonate‐based electrolyte (e.g., 1.0 m LiPF_6_ in EC/DMC (v/v, 1/1)). Normally the reason for this observation is attributed to the SEI formation on the surface of graphite (Figure 9c). However, if the SEI coated graphite (formed after cycling battery in the carbonate‐based electrolyte) is assembled in a new battery using ether electrolyte (e.g., 1.0 m LiTFSI, 0.4 m LiNO_3_ in DOL/DME (v/v, 1/1), abbreviated as 1.0 m/0.4 m), graphite exfoliation was observed, which indicated that Li^+^‐solvent co‐insertion had occurred. However, in a higher salt concentration (2.5 m/0.4 m), the graphite anode could work normally in ether, indicating that no Li^+^‐solvent co‐insertion had occurred (Figure 9d). This result indicates that the SEI layer was unable to protect the graphite anode effectively in ether electrolyte. On the contrary, the concentration of metal salts (1.0 m/0.4 m vs 2.5 m/0.4 m) had a relatively significant impact on the graphite exfoliation behavior. Furthermore, Figure 9e tested the graphite in high concentration ether electrolytes, where Figure 9f shows the electrochemical performance of SEI‐coated graphite in ether at low concentration (1.0/0.4 m) and in carbonate electrolytes. According to the comparison, the graphite anode in the low concentration ether was not stable. These observations cannot be explained by the conventional SEI theory: how can SEI‐coated graphite anode exhibit exfoliation (i.e., Li^+^‐solvent co‐insertion) if the SEI can stabilize the graphite anode? Additionally, how can the concentration of salts and solvent type used in the electrolyte have such strong influence on graphite performance (i.e., reversible Li^+^ (de‐)intercalation versus Li^+^‐solvent co‐insertion causing graphite exfoliation)?

Ming et al. further analyzed the salt concentration effect on graphite stability. The Raman S‐N‐S bending frequency in TFSI^−^ can be shifted depending on the interaction with Li^+^ ions (Figure 9g). Typically, it can be divided into a few peaks associated with different aggregation states including “free ion” (FI) (737 cm^−1^),“loose ion pair” (LIP) (741 cm^−1^), “intimate ion pair” (IIP) (745 cm^−1^), and “aggregated ion pair” (AIP) (747 cm^−1^). Obviously, higher salt concentration strengthens the interaction between salt ion pairs, leading to the formation of aggregated ion pairs. Such interaction between the cations and anions weakens the interaction between cations and solvent molecules significantly (Figure 9g). Figure 9h shows the radial distribution function (RDF) of Li ion to the oxygen of solvents (DOL and DME, the top figure) and the Li ion to the oxygen of the TFSI^–^ anions (bottom figure) with and without the addition of NO_3_^–^. The results show the DME molecules tended to occupy the first solvation shell compared with the DOL molecules. In addition, the addition of NO_3_^–^ changes the solvation structure to replace the TFSI^–^ anions. Finally, combining with the molecular dynamic simulations (Figure 9i–k), the addition of NO_3_^–^ introduced a negatively charged region, which interacted with more adjacent Li^+^ ions to form larger aggregates, in turn weakening the Li^+^‐solvent interaction.

In short, the mainstream strategies to optimize the electrolytes, such as adding additives, using multiple solvents and changing the anions have been considered to help the formation of better SEI according to the conventional SEI theory. However, the effect of solvation structure in the electrolytes should not be ignored. The internal interactions between additives and cations, cations and solvents, additives and anions, anions and cations can alter the solvation structure, which has strong effect on the electrode performance, such as the strength of Li^+^‐solvent interaction affecting the graphite performance, that is, reversible Li^+^ (de‐)intercalation versus Li^+^‐solvent co‐insertion causing graphite exfoliation.

### 5.2 Adjusting the Anions

One of the most effective ways to optimize the solvation structure is to adjust the anions in electrolytes. Strong electrostatic interaction between anions and cations leads to the formation of ion pairs, thus inhibiting the mobility of cations. Also, ion pairs can promote the formation of ion clusters, leading to a tighter solvation shell and inhibiting the de‐solvation process of cations. Thus, modulating the anions to break up the strong interaction between ion pairs serves to improve the electrochemical performance.

#### 5.2.1 Impact of Anions on Solvation Structure

**Figure 10:**
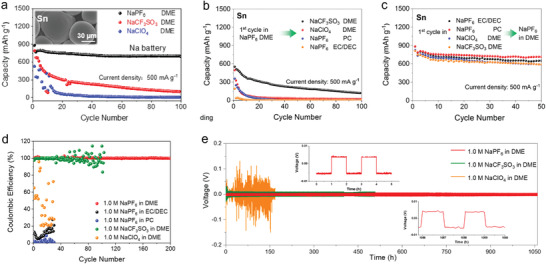
Electrochemical performance of electrolytes using different anion‐based salts. A) Cycling performance of Sn anode in 1.0 m NaPF_6_, NaCF_3_SO_3_, or NaClO_4_ in DME. b) Sn@SEI electrodes in different electrolytes. c) Sn@SEI electrodes in the 1.0 m NaPF_6_ in DME electrolyte. Reproduced with permission.^[25^ ^]^ Copyright 2020, American Chemical Society. d) Coulombic efficiency in different electrolytes. e) The voltage–time curves of Na | Na symmetrical cells in different electrolytes. Reproduced with permission.^[36^ ^]^ Copyright 2020, American Chemical Society.

In order to confirm the impact of anions on solvation structure, Ming et al. investigated the solvation behavior of different anions in SIBs.^[^ ^25^, ^36^ ^]^ First, the authors researched Sn alloy anodes in the three different anion‐based electrolytes, including NaPF_6_, NaCF_3_SO_3_, and NaClO_4_ in DME solvents. As shown in **Figure** **10**a, Sn anode displays a durable cycling performance in electrolytes containing PF_6_^–^ anions; however, the Sn anode is incompatible with electrolytes containing CF_3_SO_3_^–^ and ClO_4_^–^ anions. In addition, Figure 10b,c shows that the SEI formed on Sn anode in the NaPF_6_ electrolytes cannot protect (stabilize) the Sn anode when cycling in incompatible electrolytes. On the contrary, after undergoing several cycles in incompatible electrolytes (e.g., NaPF_6_ in PC, NaClO_4_ in DME), the Sn anode can work well (remains stable) in the compatible NaPF_6_. These results show that the electrolyte composition (e.g., solvation structure) dominates the anode performance rather than the formed SEI. Furthermore, the influence of anion type on electrochemical performance was confirmed by Na plating/stripping process in the Na | Cu half cells and Na | Na symmetrical cells (shown in Figure 10d,e). Obviously, the Sn anode demonstrates a more stable performance in electrolytes containing PF_6_^–^ anions. These electrochemical results show that electrolyte composition, especially the type of anions, has a profound impact on the electrochemical performance by changing the dominant solvation behavior.

**Figure 11:**
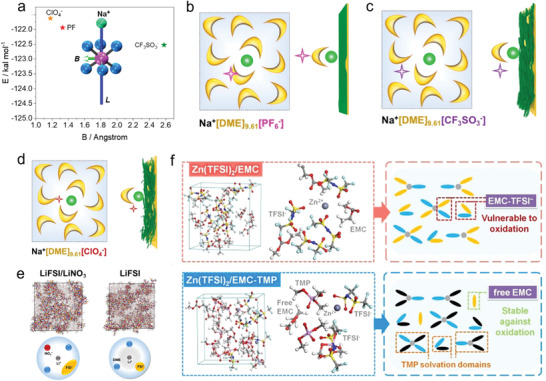
Reported examples effect of anion on the solvation structure. a) Typical model of anion, demonstrating the Na^+^‐anion/solvent binding energy (*L*) and the half distance between the adjacent anion/solvent (*B*). Anionic interfacial model describing the electrolyte‐sodium anode interface. The model for 1.0 m DME‐based electrolyte using different metal salts including b) NaPF_6_, c) NaCF_3_SO_3_, and d) NaClO_4_, respectively. Reproduced with permission.^[36^ ^]^ Copyright 2020, American Chemical Society. e) The solvation structure change induced by addition of NO_3_^−^ anions. Reproduced with permission.^[83^ ^]^ Copyright 2019, American Chemical Society. f) The influence of TMP for TFSI^−^ anions (Zn, violet; O, red; N, blue; C, gray; S, yellow; F, cyan; H, white). Reproduced with permission.^[87^ ^]^ Copyright 2020, Wiley VCH.

To this end, the specific impact of anions on solvation structure was studied further. The authors utilized two parameters to define the stability of solvation structure: i) *B* value, which reflects the half distance between adjacent ions or molecules and can describe the arrangement of the anions and solvent molecules. A higher *B* value means a looser stacking arrangement (i.e., loose binding) in the solvation shell. ii) *L*, as the binding energy between cations and anions, which denotes the strength of interaction between cations and anions. As shown in **Figure** **11**a, the ClO_4_^–^ anions show low binding energy with Na^+^ ion and small stacking volume (low *B* value). This result implies that ClO_4_^–^ anions exhibit a high degree of freedom and can move freely to escape the solvation shell, thereby inducing side reactions. In contrast, CF_3_SO_3_^–^ anions seem more confined due to the strong interaction with Na^+^ ion. In this regard, the side reactions can be decreased compared with the ClO_4_^–^. However, the compact interaction with Na^+^ ion inevitably brings CF_3_SO_3_^–^ anions to the electrode surface during the de‐solvation process. The PF_6_^–^ anions (Figure 11a) show a moderate binding energy and B volume with the Na^+^ ions by virtue of the DME solvents. Therefore, the simplified solvation structure and its arrangement near the anode surface are depicted in Figure 11b–d. Unlike the CF_3_SO_3_^–^ anions, the PF_6_^–^ anions are located at a place far away from the interface, which effectively reduces the interface side reactions and promotes the de‐solvation process. Zhang et al. also elucidated the role of NO_3_^–^ anions in Li ion solvation sheath.^[^ ^83^ ^]^ They found that the solvation structure of original FSI^−^ anions in the solvation sheath is altered by the introduction of NO_3_^–^ anions, promoting the complete decomposition of FSI^−^ and forming a stable SEI on the Li metal anode (Figure 11e).

Recently, Chen at al. also introduced the strongly electron‐donating solvent, TMP, into the TFSI‐EMC electrolyte, achieving a stable Zn/graphite cell.^[^ ^87^ ^]^ The adding of TMP can weaken the interaction between TFSI^–^ and EMC because of the preferential sequestration of anions into solvating TMP domains around the metal cations. Thus, the anions are confined far way with the electrode interface, in turn decreasing the side reactions (Figure 11f). In brief, it is now recognized that anions play critical role in the solvation process, and SEI formation theory, while important, may not be the only factor that can be used to tune electrode stability. Therefore, there is huge room to optimize the battery performance by modulating the anion behavior.

#### 5.2.2 Weakly Coordinating Anions

As mentioned in Section 2, the WCAs exhibit a significant feature of negative charge delocalization over the anions. This feature makes the coordinated bond between cations and anions much weaker. On the one hand, the weaker coordinated bond results in the higher solubility of salts with reduced lattice energy. On the other hand, from the perspective of solvation, the weaker coordination would reduce the ion pairs and favor loose solvation shells in the electrolytes. According to Riddlestone et al.,^[^ ^88^ ^]^ the design principles for a good WCA are relatively rigorous. First, the WCA charge should be low and at best univalent to weaken the electrostatic interaction with cations. In this regard, a large size anion would minimize residual coulombic attraction and facilitate dissolution in low polarity solvents. Second, the charge has to be highly delocalized over the entire entity and no basic (thermodynamics) or nucleophilic (kinetics) sites should be available, as they are typical coordination sites and might represent the first step toward ion pairing and further WCA degradation. Third, the WCA should only be constructed from chemically robust moieties to withstand partnering with very reactive cations/intermediates. Finally, the polarizability of the WCA surface should be low. These basic requirements often lead to the use of fluorinated entities as construction units of a WCA, as these typically fulfill all the requirements.

**Figure 12:**
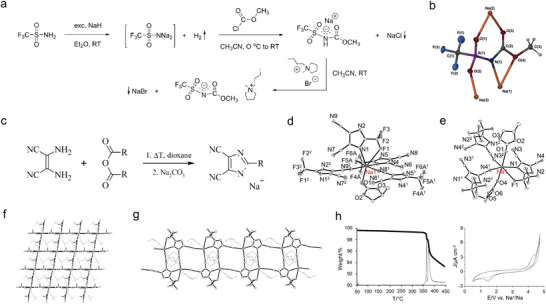
Design of new WCAs for SIBs. a) Synthesis route of Na^+^[MCTFSI]^−^. b) 3D molecular structure of Na^+^[MCTFSI]^−^ salts. Reproduced with permission.^[90^ ^]^ Copyright 2019, Wiley VCH. c) Synthesis route of Na^+^[TDI]^−^ and Na^+^[PDI]^−^, where the R represents —CF_3_ (TDI^−^) or —CF_2_CF_3_ (PDI^−^). d) Extended view of coordination environment of the sodium cation in Na^+^[TDI]^−^‐PC. e) Extended view of coordination sphere of the Na^+^ cation in Na^+^[PDI]^−^‐PC. f) View of 3D square grid supramolecular network of Na^+^[TDI]^−^‐PC projected onto the (1 0 0) plane. g) View of the 1D ladder‐like coordination polymer running along [010] direction in Na^+^[PDI]^−^‐PC. h) TG curves with derivative signal of NaTDI (left) and cyclic voltammograms of NaTDI‐PC electrolytes vs Na/Na^+^ electrodes. Reproduced with permission.^[91^ ^]^ Copyright 2014, American Chemical Society.

There are a series of WCAs which has been reported, such as the typical [PF_6_]^–^[CF_3_SO_3_]^–^, [BF_4_]^–^, and [ClO_4_]^–^. In addition, the prominent amongst alternative anions are sulfonyl substituted bis‐imides, such as [TFSI]^–^ and its smaller analogue [FSI]^–^, which have relatively favorable ion conductivity and electrochemical stability. Subsequently, a hybrid between FSI and TFSI. Other notable asymmetric sulfonyl‐imide anions are nona‐fluorobutanesulfonyl(trifluoromethanesulfonyl)imide and fluorosulfonyl(pentafluoroethanesulfonyl)imide (FPFSI).^[^ ^89^ ^]^ Recently, Gunderson‐Briggs et al. report a new asymmetric sulfonylimide anion which represents a hybrid between triflamide and carbonate, which is named methylcarbonate(trifluoromethanesulfonyl)imide (MCTFSI).^[^ ^90^ ^]^ The relative sodium salt was synthesized according to **Figure** **12**a. The three‐dimensional molecular structure diagram of the sodium salts was shown in Figure 12b. Obviously, the conformational arrangement is different from the TFSI anions which adopts a cis conformation in alkali‐metal salts, attributed to the trigonal planar carbonyl group replacing the tetrahedral sulfonyl group and the resulting unique coordination environment around Na. The proton affinity energies were further calculated, which reached −565.5 kJ mol^−1^. This is in the −650 to −550^ ^kJ mol^−1^ range on the proton affinity scale, and is similar to commonly used highly charge‐delocalized anions.

However, a challenge with the imide‐based salts is the formation of a passivation layer on Al current collectors, which complicate the synthesized process of the imide‐based salts. In 2004, Plewa‐Marczewska et al. reported two imidazole fluorine derivative sodium salts: 4,5‐dicyano‐2‐(trifluoromethyl)imidazolate (NaTDI) and sodium 4,5‐dicyano‐2‐(pentafluoroethyl)imidazolate (NaPDI).^[^ ^91^ ^]^ Compared with the imide based WCAs, the aromatic WCAs exhibit a simpler synthesis route, which is shown in Figure 12c, where the R groups represent —CF_3_ (TDI^–^) or —CF_2_CF_3_ (PDI^–^), respectively. The solvation structure of NaTDI and NaPDI in PC solvents was shown in Figure 12d and e. In Figure 12d, the coordination sphere around the Na^+^ centers comprising four TDI^–^ anions and one PC molecule. Two of the dicyanoimidazolato ligands are coordinated through the nitrogen atom of the imidazole ring, and the remaining two are bound by the cyano groups. Additionally, the solvation shell is completed by one coordinated PC molecule. Noting that the TDI^–^ anions act as a four dentate N‐donor bridging ligand linking adjacent sodium ions, which results in a metal–organic framework as shown in Figure 12f, while PC molecules occupy cavities of 3D framework. Similar with the TDI^–^ anions, the solvation structure of NaPDI in PC solvents encompasses three PDI^–^ anions and two PC molecules. Two of the PDI^–^ anions are coordinated with the cyano groups, and the third one chelates metal with the imidazole nitrogen and fluorine atoms. In addition, the PC molecules capture two coordination sites of the Na^+^ cation; in this regard, one of four imidazole donor centers remain uncoordinated, and dicyanoimidazolate anions in crystal lattice of NaPDI acts as tridentate ligand. This results in formation of ladder‐like coordination polymer propagating in the direction of the *Y* axis, as depicted in Figure 12g. PC molecules are located above and under the plain of the ladder constituting isolated rods arranged in the form of close‐packed columnar structure. Aside from the structure information, the ionic conductivity of these two salts in PC solvents was evaluated (for NaTDI: 0.5 m, 3.71 mS cm^−1^; 1 m, 3.78 mS cm^−1^ and for NaPDI: 0.5 m, 3.79 mS cm^−1^; 1 m, 3.83 mS cm^−1^). It is worth considering that the ionic conductivity is significantly low compared with the market available sodium salts, such as NaPF_6_, NaClO_4_, and NaTFSI in PC (which is between 6 and 8 mS cm^–1^ at room temperature).

In our view, regardless of NaTDI or NaPDI, the WCAs exhibit a large amount of various coordinated sites, such as ring nitrogen, cyano groups, fluorine in the substituent groups and carbonyl group oxygen in the PC solvents. Therefore, the six‐coordinated Na^+^ cations could form the complete first solvation shell. On the basis of this, it is inevitable that ion pairs will form and inhibit ionic conductivity. Nevertheless, a long‐distance framework type ordering of both TDI^−^ and PDI^−^ anions are beneficial for the structural stability and electrochemical stability, which possess a thermal stability over 300 °C and voltage window over 4.5 V (NaTDI) and 4.2 V (NaPDI) (Figure 12h).

The WCAs are one of the most effective strategies to enhance the kinetic performance, not only in the bulk electrolyte but also at the interface. Moreover, apart from the beneficial coordination properties, some research indicated that most WCAs exhibit superior oxidative stability,^[^ ^88^, ^92^, ^93^ ^]^ which allows these salts to be compatible with high voltage electrode materials. This is because the anions’ difference in solvation structure can change the interfacial model (i.e., interaction between the anion, solvent, cations and electrode) on the electrode surface.^[^ ^39^ ^]^ However, thus far, the development of new WCAs entails is progressing very slowly, due to the intricate synthesis process and multiple design requirements. Therefore, research on designing new WCAs for non‐aqueous electrolytes is needed since, despite the challenges, it has great potential.

#### 5.2.3 Anion Receptor Additives

In addition to adjusting the coordination structure and electron delocalization degree of anions, introducing anion receptor additives is another avenue to modulate the solvation structure and enhance electrochemical performance. Generally, anion receptors are a class of organic ligand that could efficiently and selectively coordinate with the anions and negatively charged functional groups like carboxylate and phosphate through *π*–*π* coordination bonds or hydrogen bonds in the electrolytes.^[^ ^94^ ^]^ The strong interaction between receptor additives and anions, weakens the electrostatic interaction between anions and cations, thereby reducing ion pair formation and improving ionic mobility. However, a one key drawback lies in the enhanced decomposition of electrolytes due to the addition of anion receptor additives; such decomposition thickens the SEI layer, and in turn damages the rate performance of metal ion batteries. Taking this into account, an improved formulation is needed to maximize the functionality of the anion receptor additive and minimize its negative effect on the rate performance.

**Figure 13:**
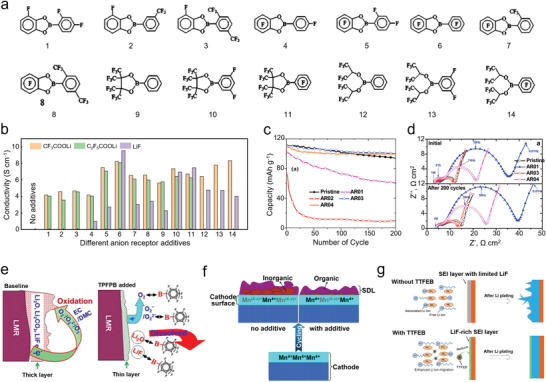
Selected examples on using anion acceptors in LIBs. a) Chemical structure of boronate‐based anion receptors. b) Conductivity summary of three salts with the addition of different additives. Reproduced with permission.^[^ ^98^ ^]^ Copyright 2004, The Electrochemical Society, Inc. c) Cycle performance with different anion receptor additive adding. d) The corresponding EIS plots of (c). Reproduced with permission.^[^ ^94^ ^]^ Copyright 2010, American Chemical Society. e) Scheme of the functioning mechanism of TPFPB: (left) Thick passivation layer formation in baseline electrolyte; (right) significantly reduced passivation layer formation in TPFPB added electrolyte. Reproduced with permission.^[^ ^95^ ^]^ Copyright 2014, Elsevier B.V. f) Schematic diagram of the cathode and electrolyte interphase with and without the additive. For simplicity, only Mn cations are shown on the cathode surface. Reproduced with permission.^[^ ^97^ ^]^ Copyright 2017, American Chemical Society. g) Schematic illustration of the structural changes of Li metal anodes and property of electrolyte: (up) without TTFEB; (down) with TTFEB. Reproduced with permission.^[^ ^99^ ^]^ Copyright 2018, Elsevier B.V.

Although there have been some reports on using anion receptor additives in LIBs,^[^ ^95^, ^96^, ^97^ ^]^ there are currently no reports on using anion receptor additives in SIBs. Therefore, we use the LIB as an example to clarify this idea. The boron‐based anion acceptor additives are the most widely used in LIBs due to their higher fluoride affinity to coordinate with the fluorine atoms in the commercial PF_6_^–^, TFSI^–^, and FSI^–^. Lee et al. did substantial work related to the boron‐based anion acceptor additives, including exploring the new functional anion acceptor additives, investigating the impact of additives on ionic conductivity, and the negative effect on interface resistance.^[^ ^98^ ^]^ For example, Lee and his collaborators synthesized a series of boronate compounds with different fluorinated aryl and fluorinated alkyl groups, shown in **Figure** **13**a. When these receptors were used as additives in DME solvents, a striking increase of ionic conductivity was obtained.^[^ ^98^ ^]^ The conductivity of three different lithium salts (i.e., LiF, CF_3_COOLi, and C_2_F_5_COOLi) was studied with the same additive concentration, as shown in Figure 13b. It is worth noting that the LiF salts are almost insoluble in bare DME solvents. As for the CF_3_COOLi and C_2_F_5_COOLi salts, the addition of anion receptor additives significantly improves the conductivity of the solution by two orders of magnitude. They further showed that the degree of complexation is closely related to the structures of the fluorinated aryl and alkyl groups, which act as electron‐withdrawing groups. Despite the fact that the conductivity was enhanced systematically, the electrolyte decomposition was accelerated using these additives.

Qin et al. investigated the electrochemical performance of four different anion receptor additives, including 2‐(2,4‐difluorophenyl)tetrafluoro‐1,3,2‐benzodioxaborole (AR01), 2,5‐bis(trifluoromethylphenyl)tetrafluoro1,3,2‐benzodioxaborole (AR02), tris(1,1,1,3,3,3‐hexafluoroiso‐ propyl) borate (AR03), and bis(1,1,1,3,3,3‐hexafluoroisopropyl)pentafluorophenylboronate (AR04).^[^ ^94^ ^]^ Figure 13c displays the discharge capacity of assembled LIBs that were cycled between 3.0 and 4.0 V with a constant current of 1C at room temperature. The baseline electrolyte was 1.2 m LiPF_6_ in EC/EMC (3:7). It was obvious that the addition of AR01 and AR04 caused drastic decrease of capacity compared to the case without anion receptor additives. For AR03 and AR04 additives, a slight capacity enhancement was observed. Electrochemical impedance analysis showed that the AR01, AR02, and AR04 additives can significantly enlarge the impedance, especially AR02 which increased impedance by more than ten times (not shown in the Figure 13). In addition, the anion receptors mostly affected the low‐frequency semicircle (in the range of tens of hertz) in the impedance spectra, which is related to the charge transfer reaction at the SEI (Figure 13d). These results show that accelerated decomposition of electrolyte can cause thicker SEI thicken. Therefore, careful balance between increased conductivity and increased interface resistance must be maintained when adding anion additives.

Recently, some new anion receptor additives also were explored, such as tris (2, 2, 2‐trifluoroethyl) borate (TTFEB),^[^ ^97^ ^]^ superhalogen based anion receptors (B[C_2_HBNO(CN)_2_]_3_,^[^ ^96^ ^]^ B[C_2_HBNS(CN)_2_]_3_,^[^ ^96^ ^]^ and B[C_4_H_3_BN(CN)_2_]_3_),^[^ ^96^ ^]^ tris(pentafluorophenyl)borane ((C_6_F_5_)_3_B, TPFPB), etc. For instance, Zheng et al. proposed that TPFPB as an anion additive. They showed that it could effectively confine the highly active oxygen species released from structural lattice of the Li‐rich, Mn‐rich layered composite, attributed to its strong coordination ability and high oxygen solubility (Figure 13e).^[^ ^95^ ^]^ Such coordinated structure mitigated the electrolyte decomposition caused by the oxygen species attack, in turn reducing the amount of byproducts on the cathode surface. Additionally, the inorganic products (Li_2_O and LiF) were soluble in TPFPB containing electrolytes, which caused only a thin layer on the surface of cathode, reducing the interfacial resistance.

Similarly, Ma et al. utilized a new boron‐based anion receptor (TTFEB), as an electrolyte additive in cells containing an Li‐ and Mn‐rich layered oxide cathode, Li_1.16_Ni_0.2_Co_0.1_Mn_0.54_O_2_.^[^ ^97^ ^]^ In the presence of only a small amount of TTFEB additive, LiF and Li_2_CO_3_ were reduced, yielding phosphorus‐containing species such as LiP*_x_* F*_y_* and Li*_x_* PO*_y_* F*_z_*. This likely promotes more free movement of Li ions and electrons, better utilization of the cathode material, and overall higher capacity. In addition, the reduction of tetravalent Mn^4+^ at the cathode surface was also minimized, as shown in Figure 13f. Subsequently, the TTFEB anion receptor additive was applied in a lithium metal battery.^[^ ^99^ ^]^ Due to the strong coordination with the PF_6_^–^ anions, the lithium transference numbers could be enhanced significantly. The increased lithium ions transference numbers on the one hand induced a larger Sand's time *τ*, suggesting longer lifetime of the cell before the Li dendrite growth that enable uniform Li deposition on the Li metal anode. On the other hand, such high lithium ions mobility was beneficial for the rate performance of batteries. Additionally, the fluorinated groups participated in the forming of LiF‐rich SEI layer on the Li metal surface to some extent, in turn spurring the uniform dendritic growth of metal lithium (Figure 13g).

In short, the interaction between the anions and cations can be modulated by adding anion receptor additives. The altering of solvation structure markedly enhances the ion mobility of cations, in turn improving the electrochemical performance. Furthermore, the fluorinated groups in the anion receptors are beneficial for the favorable SEI to inhibit the metal dendrite. However, there are still some issues underlying on the anion receptor additives, such as the accelerated electrolyte decomposition. Additionally, the detailed mechanism of optimizing solvation structure originating from anion receptor additives needs is not fully understood, especially from the perspective of interface arrangement and de‐solvation process.

### 5.3 Adjusting the Solvent Molecules

Regulating the solvent molecules seems to be a more straightforward strategy to optimize the solvation structure thanks to the dominant position of solvent molecules in the first solvation shell. The coordination ability of solvent molecules targeting the cations basically determines the de‐solvation process. Generally, a strong coordination interaction (or dipole) inevitably leads to cation‐solvent co‐intercalation such as Li^+^‐solvent in the graphite anode (usually described as the electrolyte incompatibility). The cation‐solvent co‐insertion is highly undesirable because it leads to structure destruction of electrode materials (i.e., graphite exfoliation) resulting in degraded electrochemical performance. Furthermore, the intercalated solvent molecules in solid phase are decomposed upon the charge/discharge process, releasing gases and aggravating the side reactions, in turn significantly damaging the battery performance. Nevertheless, a very weak interaction between cations and solvents is also harmful to battery performance due to the increased probability of ion pair formation, thereby precluding cation migration in the bulk electrolytes.

#### 5.3.1 Impact of Solvents on Solvation Structure

**Figure 14:**
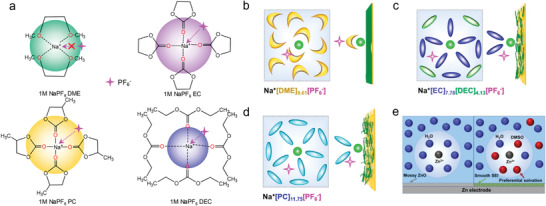
The solvation behavior of different solvent‐based salts in SIBs. a) Proposed coordination structure of Na^+^‐solvents pairs, in which the opportunities of PF_6_^−^ to contact the Na^+^ were presented. b) The interface model of Na^+^‐DME‐PF_6_^−^, c) Na^+^‐EC/DEC‐PF_6_^−^, and d) Na^+^‐PC‐PF_6_^−^. Reproduced with permission.^[25^ ^]^ Copyright 2020, American Chemical Society. e) The solvation structure of Zn^2+^ with DMSO adding. Reproduced with permission.^[100^ ^]^ Copyright 2020, American Chemical Society.

The impact of different solvents on cation solvation structure was first investigated by Ming et al.^[^ ^25^, ^36^ ^]^ Combining simulations and spectrum analysis, they proposed a coordination model to explain the reason that NaPF_6_ is only compatible in DME solvent but not EC, PC, or DEC solvents. As shown in **Figure** **14**a, the NaPF_6_ in DME shows a stable solvation structure. This means that PF_6_^−^ anions have adequate freedom of movement, and the PF_6_^−^ does not easily make contact with the Na^+^ ion to form ion pairs because of the bidentate chelation of Na^+^‐DME. In contrast, the PF_6_^−^ is relatively easy to make contact with Na^+^ because of the lower steric hindrance resulting from the monodentate chelation of Na^+^‐EC or Na^+^‐PC, even though the distance of Na^+^ and PF_6_^−^ might be somewhat large. Such results are in line with the discussion presented in Section 5.2.1. The different solvents exhibit distinct coordination ability with the cations, which causes a change in anion position as well as the probability of ion pair formation. Therefore, as depicted in Figure 14b–d, the DME solvents keep the PF_6_^−^ anions far from the cations to avoid interface adsorption, thereby reducing the anion decomposition and side reactions. In contrast, the EC, PC, DEC solvents have limited ability to retard the anion migration near the interface, resulting in electrolyte incompatibility. Therefore, the solvent affects the compatibility of the electrolyte to a large extent because it affects the behavior of anions during the de‐solvation process. However, there are not many reports on the influence of different solvent types on solvation structure in SIBs, and more in‐depth research is needed to further understand the effect of solvents on solvation structure and the related electrochemical behavior.

#### 5.3.2 Multiple Solvents

Early efforts to optimize solvent components mainly involved adjusting the solvent type, typically combining various solvents to form binary and ternary solvents. According to Bommier et al.,^[^ ^53^ ^]^ the most commonly used sodium ion battery electrolyte is EC/DEC. Due to the complementary advantages of the two solvents (high dielectric constant of EC and low viscosity of DEC), these solvent mixtures can achieve a favorable electrochemical performance. Unfortunately, there are few reports that explain the solvation process in SIBs in the presence of multiple solvents. Recently, Cao et al. proposed a solvation mechanism to explain the advantage of using binary DMSO/H_2_O solvents in aqueous Zn ion battery.^[^ ^100^ ^]^ As shown in Figure 14e, the DMSO solvents partially replace the H_2_O molecules in the first solvation shell due to the higher Gutmann donor number of DMSO (29.8) compared to that of H_2_O (18). The preferential solvation of DMSO with Zn^2+^ and strong H_2_O‐DMSO interaction inhibit the decomposition of solvated H_2_O. In addition, the decomposition of solvated DMSO is beneficial for the formation of good SEI. These results showed that utilizing multiple solvents can be an effective strategy to optimize the solvation structure, in turn achieving the favorable electrochemical performance.

#### 5.3.3 Adjusting the Amount of Solvents

**Figure 15:**
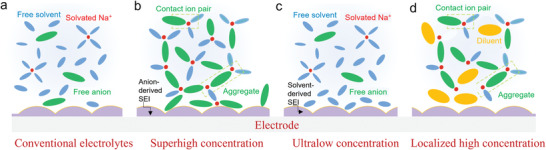
Solvation and interface model for different electrolyte concentration. a) Conventional electrolytes (1 m). b) Superhigh concentration electrolytes. c) Ultralow concentration electrolytes. d) Localized high concentration electrolytes.

In addition to using multiple solvents, another widespread strategy to optimize solvation behavior and battery performance, is by adjusting the number of solvent molecules. The number of solvent molecules can be changed by tuning the concentration. As shown in **Figure** **15**a, the conventional electrolytes (usually use 1 m concentration) maintain a balanced number of anions and solvents, which can achieve a favorable ionic conductivity and a moderate voltage. However, side reactions can occur at high voltage elevation because of the decomposition of free anions and solvents near the electrode and electrolyte interface. As the electrolyte concentration is increased (Figure 15b), the solvation structure of Na^+^ ion changes. Due to the insufficient number of solvent molecules, the cations and anions tend to from ion pairs and anion aggregation occurs. The reduction of the number of free solvent molecules extends the potential window due to a decreased solvent decomposition near the interface. In addition, if we considered the de‐solvation process of Na^+^ ions, the highly concentrated electrolytes improve the diffusion process of Na^+^ because there is little solvent co‐intercalation. Likewise, the SEI is mainly composed of decomposition products of anions unlike the conventional electrolytes. Although the highly concentrated electrolytes display an extended voltage window and fast de‐solvation process, their high cost and limited ionic conductivity (high viscosity) are major limitations. Therefore, an opposite strategy of using ultralow concentration electrolytes has been proposed.

As shown in Figure 15c, the dilute electrolytes exhibit sufficient number of free solvent molecules and the solvation process of salts occurs easily. Such dilute electrolytes effectively decrease the cost and extend the working temperature range of batteries. But due to the surplus of free solvent molecules, the potential window is reduced. Moreover, the cation‐solvent co‐intercalation is hard to avoid. Therefore, localized high concentration electrolytes have been proposed as a compromise. As depicted in Figure 15d, an inert solvent is introduced to “dilute” the concentrated electrolyte without changing the local coordination environment of the concentrated electrolytes. Such strategy not only decreases the total cost and electrolyte viscosity, but also maintains the local high concentrated coordination environment, achieving a high voltage window and stable electrochemical performance. In the next section, some examples of these different electrolytes are discussed.

##### Superhigh Concentration Electrolytes

**Figure 16:**
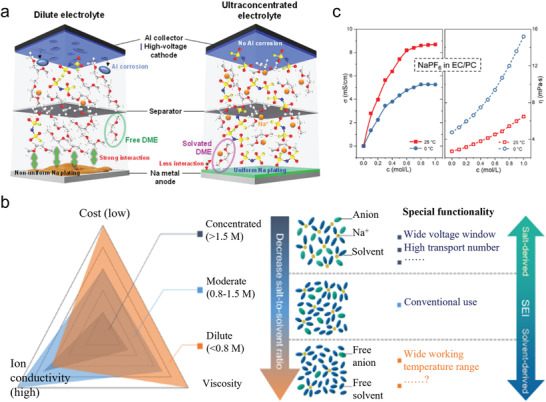
Illustration of superhigh and ultralow concentration electrolytes. a) Solvation behavior and interface model in the electrolyte of 5 m NaFSI in DME. b) The varied physical and chemical properties of electrolytes changed by the concentration. c) The changed conductivity and viscosity by decreasing the concentration. Reproduced with permission.^[111^ ^]^ Copyright 2020, American Chemical Society.

Superhigh‐concentration electrolytes have attracted great attention in recent years since an improved performance can be obtained by employing such electrolyte technology. The earliest reports of superhigh‐concentration electrolytes date back to 1985. McKinnon and Dahn showed that co‐intercalation of Li^+^‐PC into ZrS_2_ layered material can be avoided by using a saturated electrolyte; in contrast, they observed significant co‐intercalation in 1 m electrolyte.^[^ ^101^ ^]^ In 2003 Jeong et al. found that graphite can be compatible with PC solvents as long as the electrolyte concentration was enough high.^[^ ^102^ ^]^ However, the earliest research on using highly concentrated electrolytes in SIBs was only reported in 2014 by Terada et al. investigated the properties of tetraethylene glycol dimethyl ether (tetraglyme/TEGDME) and NaTFSI mixtures. The authors revealed the formation of a “solvate ILs” in which [Na(tetraglyme)]^+^ cationic charge carriers are formed for equimolar ratio of NaTFSI:tetraglyme. The prepared electrolyte exhibited an ionic conductivity of 0.61 mS cm^−1^ (at 30 °C) and an electrochemical window stability of 4 V versus Na/Na^+^.^[^ ^103^ ^]^ Guo et al. used a high‐concentration 4 m NaFSI/triglyme as electrolyte to fabricate SIBs with excellent performance.^[^ ^104^ ^]^ In 2017, Lee at al. presented an ultraconcentrated electrolyte composed of 5 m NaFSI in DME for Na metal anodes coupled with high‐voltage cathodes.^[^ ^35^ ^]^ As shown in **Figure** **16**a, the compact interactions between solvent molecules and Na^+^ ions could decrease the number of free DME molecules dramatically, in turn inhibiting Na dendrite formation. Many studies followed in which highly concentrated electrolytes such as NaFSI‐diglyme,^[^ ^105^ ^]^ NaFSI‐DME,^[^ ^106^ ^]^ NaFSI‐succinonitrile,^[^ ^107^ ^]^ NaFSI‐DMSO,^[^ ^108^ ^]^ and NaFSI‐TMP were reported.^[^ ^109^ ^]^ Several recent reviews have appeared covering the concept of superhigh concentration electrolytes.^[^ ^32^, ^33^, ^34^, ^110^ ^]^ Although the superhigh concentration electrolytes have shown great advances in battery voltage and diffusion behavior, the high cost and high electrolyte viscosity issues remain unsolved.

##### Ultralow Concentration Electrolytes

Decreasing the concentration of electrolytes is an effective approach to reduce battery cost. Different from Li^+^ ions, Na^+^ ions exhibit smaller Stokes radius and de‐solvation energy; thus, it is possible for SIBs to employ low‐concentration electrolytes to obtain enough kinetics performance. Recently, Li et al. proposed an unusual ultralow‐concentration electrolyte for SIBs to further reduce the cost and expand the working temperature range. Their favorable performance benefited from the low viscosity of a dilute electrolyte and the formed organic‐dominated solid electrolyte interphase.^[^ ^111^ ^]^ As shown in Figure 16b, the authors summarized the change of physicochemical properties showing that with decreasing electrolyte concentration, the cost, ion conductivity, and viscosity improved.

The ion conductivity and viscosity in different concentrations of electrolytes are given in Figure 16c. It can be seen that the ionic conductivity exhibits a downward‐parabola‐like relationship with concentration; in other words, the higher the concentration is, the slower the rate of increase in conductivity. At lower temperature, the change in conductivity becomes smaller. More importantly, the viscosity increases dramatically with concentration at a lower temperature. This result implies that high concentration electrolytes suffer from high viscosity at lower working temperature. Therefore, we can conclude that the ultralow concentration electrolytes benefit SIB performance at low temperature. The sufficient number of free solvent molecules make it possible to maintain a moderate electrolyte viscosity even at extreme temperatures. However, the poor ion conductivity is still the issue due to the limited number of free carriers.

##### Localized High Concentration Electrolytes

**Figure 17:**
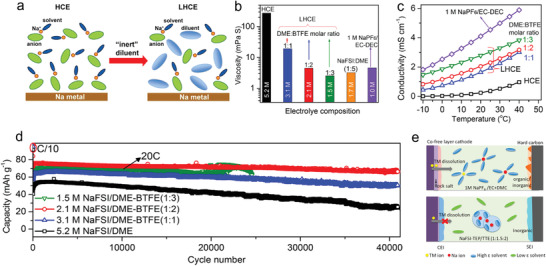
Solvation behavior and related electrochemical performance in LHCE. a) The solvation and interface model, b) conductivity, c) viscosity, and d) electrochemical performance of SIBs in LHCE. Reproduced with permission.^[112^ ^]^ Copyright 2018, American Chemical Society. e) The design principle of nonflammable localized high concentration electrolytes. Reproduced with permission.^[113^ ^]^ Copyright 2020, American Chemical Society.

The first attempt using localized high concentration electrolytes (i.e., LHCE) in SIBs was conducted by Zheng et al.^[^ ^112^ ^]^ They effectively decreased the electrolyte concentration to less than 1.5 m by adding a hydrofluoroether as an “inert” diluent. As shown in **Figure** **17**a, the “inert” diluent has minimal or no effect on the solvation structure of cation−anion aggregates that exist in concentrated electrolyte. Instead, it can significantly lower the sodium salt concentration, reduce the viscosity (Figure 17b), increase the conductivity (Figure 17c), and improve the wettability of the electrolyte. Due to the above advantages, the adding of diluents make the Na | Na_3_V_2_(PO_4_)_3_ battery show a favorable cyclic stability (Figure 17d). Recently, Jin et al. reported a nonflammable LHCE (sodium bis(fluorosulfonyl)imide‐triethyl phosphate/1,1,2,2‐tetrafluoroethyl‐2,2,3,3‐tetrafluoropropyl ether (1:1.5:2 in molar ratio)) for highly reversible SIBs (Figure 17e).^[^ ^113^ ^]^

Although the LHCE meet the requirements of low cost and low viscosity and maintaining the advantages of high concentration electrolytes, the available component candidates are limited. Yamada et al. clarified the requirements for the inert diluents in a recent review.^[^ ^32^ ^]^ They include: 1) low viscosity that can effectively lower the viscosity of the entire electrolyte; 2) low cost that can decrease the overall cost of the electrolyte; 3) appropriate permittivity and coordination property that enable a high solubility of the concentrated electrolyte, while not changing the local coordination environment of the concentrated electrolyte; 4) sufficient inertness/stability that does not compromise the electrochemical window of the concentrated electrolyte; and 5) non‐flammability and low volatility that do not compromise the safety of the concentrated electrolyte. Therefore, it is essential that suitable inert diluents are identified for effective LHCE in SIBs.

In this section, two classical theories are discussed, which encompass the SEI theory and solvation theory. Further, we summarized the possible strategies to modulate the solvation structure and interface model to achieve optimized electrochemical performance in SIBs. One is adjusting the anions, such as designing different weakly coordinating anions to meet ideal crystallographic properties and performing anion acceptors to change the coordination environment of anions. A second avenue is to modulate the solvent molecules by mixing multiple solvents whose functionalization is different. Adjusting the number of solvent molecules is yet another effective and way. Different number of solvent molecules can be achieved by modulating electrolyte concentration. The different concentrations induce changes in the solvation structure and interface arrangement.

## 6 Characterization Methodology of the Solvation Structure

Due to the abstract nature of solvation structures, characterization techniques for direct visualization are not yet mature. However, with the development of spectroscopic techniques as well as the theoretical simulation, deciphering the solvation structure is becoming more feasible. Therefore, in this section, we focus on the applications of some spectroscopic techniques in the field of solvation design, such as Fourier transform infrared spectroscopy (FTIR), Raman spectroscopy, and nuclear magnetic resonance (NMR). In addition, the theoretical calculation methods commonly used in analyzing solvation structures are also briefly introduced.

### 6.1 Spectroscopic Techniques

**Figure 18:**
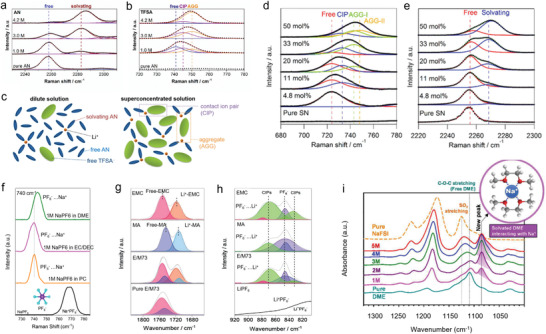
Raman and FTIR characterization of solvation structure. a) Raman spectra of LiTFSA/AN solutions in 2230−2310 cm^−1^ (C≡N stretching mode of AN molecules) and b) 720−780 cm^−1^ (S—N stretching, C—S stretching, and CF_3_ bending mode of TFSA^−^). Points and solid lines denote experimental spectra and fitting curves, respectively. c) Representative environment of Li^+^ in a conventional dilute solution (i.e., ≈1 mol dm^−3^) and a salt‐superconcentrated solution (i.e., 4.2 mol dm^−3^). Reproduced with permission.^[114^ ^]^ Copyright 2014, American Chemical Society. d) Raman spectra of the NaFSA/SN solutions at various concentrations in the regions of 680−780 cm^−1^ (vibrational mode of FSA−) and e) 2220−2300 cm^−1^ (C≡N stretching mode of SN) at room temperature. The points and lines show the experimental spectra and fitting curves, respectively. Reproduced with permission.^[107^ ^]^ Copyright 2017, American Chemical Society. f) Raman spectra of PF_6_^−^ anions in different kinds of solvents. Reproduced with permission.^[39^ ^]^ Copyright 2020, American Chemical Society. g) FTIR spectra of Li^+^ in different kinds of solvents. h) FTIR spectra of PF_6_^−^ and Li^+^–solvent–PF_6_^−^ complexes (CIPs). Reproduced with permission.^[39^ ^]^ Copyright 2021, Wiley‐VCH. i) FTIR spectra of *x* m NaFSI‐DME (*x* =0,1, 2,3,4, or 5) and pure NaFSI salt. Reproduced with permission.^[35^ ^]^ Copyright 2017, American Chemical Society.

The spectroscopic techniques are useful ways to investigate the interaction between various electrolyte components. The coordination state on different atoms can be detected by the change of electron cloud density or bond vibration. Raman spectra have been widely employed to investigate the interactions between solvent molecules, cations, anions, and additives. As shown in **Figure** **18**a, Yamada et al. reported unusual reductive stability of a superconcentrated acetonitrile (AN) electrolyte. They found the C≡N stretching peak dependency on different AN coordination states.^[^ ^114^ ^]^ Pure AN shows a C≡N stretching band (v_2_ mode) at 2258 cm^−1^ derived from free AN molecules (i.e., without coordinating to Li^+^). With the adding of salt, another v_2_ band appears at 2282 cm^−1^ arising from Li^+^‐solvating AN molecules. Increasing the salt concentration, the free AN molecules are reduced while the coordinated AN molecules increase. In addition, turning to the vibration mode of TFSA^−^(i.e., S—N stretching, C—S stretching, and CF_3_ bending) in Figure 18b, a deconvolution analysis shows that the Raman band consists of three peaks at 740, 745, and 750 cm^−1^, arising from free anions, contact ion pairs (CIPs, TFSA^−^ coordinating to a single Li^+^ cation), and aggregates (AGGs, TFSA^−^ coordinating to two or more Li^+^ cations), respectively (Figure 18c). Therefore, the relationship between different electrolyte concentration and anion aggregated state can be deciphered clearly by Raman spectroscopy. Subsequently, Takada et al. utilized Raman analysis to investigate the solvation behavior in a superconcentrated sodium ion battery electrolyte.^[^ ^107^ ^]^ They designed a 50 mol % sodium bis(fluorosulfonyl)amide (NaFSA)/succinonitrile (SN) electrolyte, which enabled highly reversible Na^+^ insertion into a hard carbon negative electrode without any electrolyte additive. The Raman spectrum of such electrolyte shows that the of FSA^−^ anions band shifts to higher wavenumber when the anion is coordinated with one, two, or three cations to form contact ion pairs (CIPs), aggregates‐I (AGGs‐I), or AGG‐II, respectively. In addition, the coordination state of SN is changed with the increase of concentration (Figure 18d,e). The Raman band ≈2256 cm^−1^ is assigned to the C≡N bond of the SN solvent and is shifted to higher wavenumbers when the C≡N group is coordinated with an Na^+^ cation. Hence, Raman spectroscopy can clearly reveal the coordinated situation of anions and solvent molecules in the bulk electrolytes. In addition to explore the relationship between solvation structure and salt concentration, Raman spectroscopy is also used to detect the coordination environment of cations/anions in different solvents. Zhou et al. confirmed that the cation solvation structure, particularly the type and location of the anions in the electrolyte, plays a critical role in SIB alloying anode stabilization.^[^ ^25^ ^]^ The interactions in different type of anions and solvents are investigated by Raman spectroscopy (Figure 18f).They found a redshift of the P—F vibration in the PF_6_^−^ anion from 768 to 740 cm^−1^ during the dissolution process, which represents the solvation process of Na^+^. At the same time, The PF_6_^−^ anions have a high degree of mobility in EC/DEC and PC compared to DME, as confirmed by the higher wavenumber in DME (at 743 cm^−1^) compared to 740 cm^−1^ in EC/DEC and PC. Therefore, Raman analysis is the most widely employed method for deciphering the solvation structure related interactions due to the high sensitivity and clear scattering peaks. Aside from the organic electrolyte (LIBs, SIBs, etc.), it also has been widely implemented in the field of aqueous electrolytes to explain the different solvation behaviors.^[^ ^115^, ^116^, ^117^, ^118^ ^]^

FTIR spectroscopy is an alternative to Raman to investigate the solvation structure in organic electrolytes. Similar to the Raman technique, it reflects the bond vibration, in turn exploring the interactions between cations, anions, and solvent molecules. However, the absorption peak of the target functional group is often interfered by some other organic groups in similar positions, making it hard to detect the change in vibration. Therefore, the choice of Raman or FTIR must be decided according to the actual situation of the electrolyte system. For instance, Zou et al. reported a new carbonate‐based high‐voltage electrolyte employing a mixture of ethyl methyl carbonate (EMC) and methyl acetate (MA) solvents without adding any additive. The Li^+^ ion solvation structure was studied by FTIR in detail, as shown in Figure 18g.^[^ ^39^ ^]^ They found that the combined peak at 1750 and 1747 cm^−1^, corresponding to the C═O stretch vibrations of EMC and MA, had a redshift and was split into two main peaks at 1718 and 1712 cm^−1^ when the 1.2 m LiPF_6_ salt was dissolved into the solvent mixture. It represents the solvation process of Li^+^ ions, where the Li^+^ ions coordinate with the solvents by the Li^+^—O interactions. Moreover, the solvated anion (PF_6_^−^) can be classified into uncoordinated (free) PF_6_^−^ (at 845 cm^−1^) and Li^+^—PF_6_^−^ (i.e., contact ion pairs, CIPs) at the peaks of 834 and 870 cm^−1^ (Figure 18h). This result is similar to the Raman spectrum which can reveal the aggregated state of anions in different solvents. In particular, the coordinated number or proportion of each electrolyte species can be quantitatively estimated by deconvoluting the FTIR spectra. In addition, Lee at al. confirmed the solvation structure of NaFSI‐DME electrolytes as a function of NaFSI salt concentration by FTIR.^[^ ^35^ ^]^ The characteristic C—O stretching band of pure DME is located at 1110 cm^−1^ (Figure 18i). Notably, the introduction of NaFSI into DME produced a new peak at 1085 cm^−1^, which indicates that the ion−dipole interactions between the C—O—C moieties and Na^+^ ions affect the C—O—C stretching vibration mode of DME with DME solvating Na^+^ ions sufficiently to prevent ion pairing. The peak intensity at 1085 cm^−1^, which arises from coordination between C—O—C moieties and Na^+^ ions, gradually increased as a function of NaFSI salt concentration.

**Figure 19:**
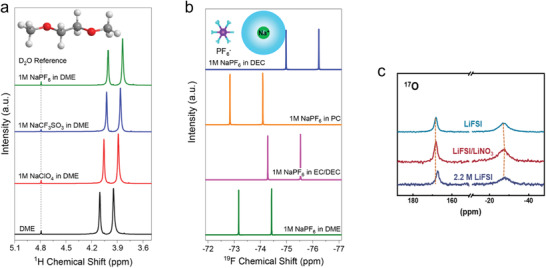
NMR characterization of solvation structure. a) ^1^H NMR spectra of different types of sodium salt in DME. b) ^19^F‐NMR spectra of PF_6_ anions in different kinds of solvents. Reproduced with permission.^[25^ ^]^ Copyright 2020, American Chemical Society. c) Natural abundance ^17^O NMR spectra of various electrolytes measured at 50 °C. Reproduced with permission.^[^ ^83^ ^]^ Copyright 2019, American Chemical Society.

Aside from Raman and FTIR, NMR is an important and effective method to study electrolyte solvation behavior. It can provide information on the number of nuclei, their chemical environment and geometry. Especially for the light elements (like the H, C, O, F, etc.), the interaction strength between atoms can be quantitatively expressed which is suitable for the investigation of electrolyte solvation behavior. As shown in **Figure** **19**a, the 1H NMR of CH_3_/CH_2_ peaks in DME shows that the 1 m NaPF_6_ in DME exhibits the most obvious chemical shift compared to NaClO_4_ and NaCF_3_SO_3_.^[^ ^25^ ^]^ This means the PF_6_^−^ anions show better coordination with DME molecules compared with ClO_4_^−^ and CF_3_SO_3_^−^ anions. In addition, the ^19^F‐NMR of PF_6_^−^ anions in Figure 19b can give the coordination situation between PF_6_^−^ anions and different solvents.^[^ ^25^ ^]^ As we know, the chemical shift reflects the extranuclear electron cloud density. If the electrons are enriched outside the nucleus, there will be a shielding effect, resulting in a lower chemical shift. For the PF_6_^−^ anions in DME and PC solvents, the strong coordination between anions and solvents would lead the chemical shift lower. On the contrary, coordination between the branched/linear DEC and the cations is not so condensed, leading to the peak shift toward higher magnetic field. Thus, we can see a medium ^19^F‐NMR peak shift in the mixed EC/DEC solvent. In addition to the^1^H spectrum and ^19^F spectrum, there are some applications of the^17^O spectrum if the anions are oxygen‐containing. For instance, Zhang et al. modulated the solvation structure of Li^+^ ions by adding the NO_3_^−^ anions into the electrolyte.^[^ ^83^ ^]^ They confirmed the change of Li^+^ ion solvation structure by the^17^O NMR combined with the theoretical simulation. As shown in Figure 19c, the chemical shifts at around 168.0 and −26.0 ppm in LiFSI electrolyte are assigned to the sulfonyl oxygen atoms (O_FSI−_)of FSI^−^ and the ethereal oxygen atoms (O_DME_) of DME, respectively. More FSI^−^ anions participate in the solvation sheath in 2.2 m LiFSI due to decreased free solvents, leading to a decreased chemical shift. However, when 0.2 m NO_3_^−^ is introduced to 2.0 m LiFSI electrolyte, the peak of O_FSI−_ (168.0 ppm) only shifts upfield by −0.2 ppm. Under the same FSI^−^/DME ratio in LiFSI/LiNO_3_ electrolyte, a higher Li^+^/FSI^−^ ratio induces a smaller shift of the O_FSI−_ peak compared with LiFSI electrolyte, indicating that NO_3_^−^ is involved in solvation instead of more FSI^−^ anions.

### 6.2 Theoretical Simulations

Although the spectroscopic characterization can give qualitative interactions inside the solvation structure, it only reflects the strength of the interactions in bulk electrolytes. However, sometimes we need to consider the local coordination environment of the electrolyte components and interface situation. Hence, theoretical simulation renders an effective avenue to analyze the solvation structure. Typically, the most common use is the radial distribution function (RDF) in the field of solvation structure.^[^ ^119^, ^120^ ^]^ It is denoted by *g*(*r*), which defines the probability of finding a particle at a distance *r* from another tagged particle. By virtue of RDF, the solvation structure of cations can be determined, including the coordinated number, the occupancy of anions, and the occupancy of additive molecules. Derived from RDF, the potential mean force (PMF) is another effective way to express the ability to obtain solvent molecules or anions into the solvation layer.^[^ ^121^, ^122^, ^123^, ^124^ ^]^ The coordination strength and energy barrier can be obtained by the contact minimum in PMF curve. In addition, Buried Volume can reflect the steric hindrance and interactions in the solvation structure.^[^ ^125^ ^]^ Aside from the simulations by molecule dynamics, the electronic structure of solvated ions is conducted by projected partial density of states (PDOS), from which we can get the information about energy level and orbitals, in turn deciphering bonding information.^[^ ^126^ ^]^ In addition, thermodynamic parameters, such as desolvation energy, binding energy, HOMO/LUMO, etc., also are used to confirm the solvation behavior.^[^ ^26^, ^39^, ^127^ ^]^

Briefly, this section introduced the common methods used to characterize the solvation structure, including FTIR, Raman, NMR, and theoretical simulations. Actually, the analysis of solvation behavior requires us to combine spectroscopy, theoretical simulations as well as experimental electrochemical performance. However, until now we have not yet been able to visualize the solvation structure. In addition, the dynamic evolution of solvation structure cannot be observed directly. Therefore, there is still a huge potential advanced characterized techniques for observing and analyzing solvation structure. For example, emerging methods such as ultrafast femtosecond mid‐FTIR spectroscopy and pulsed‐field gradient NMR analysis may help study solvation structure dynamics.^[^ ^122^ ^]^

## 7 Conclusion and Perspectives

Thanks to intensive efforts over the past decade, SIBs have become the battery system closest to commercialization after LIBs. However, like other alkali metal ion batteries, the electrolyte compatibility principle in SIBs is still not fully understood. The reason for this is the intricate interactions among the various electrolyte components. Therefore, electrolyte engineering and development have lagged behind electrode material development. For instance, the most commonly used electrolyte in SIBs is still inherited from the electrolyte design used in LIBs. No major breakthrough has taken place in the last decade. Recently, the reemergence of “solvation theory” is helping us to better understand the principles of electrolyte design in SIBs. The role of anions, solvents, and additives in the resultant solvation shell (not only SEI) is used to optimize electrolyte and SIB performance. Hence, we have summarized the recent developments in electrolytes solvation behavior particularly in SIB. First, the requirements of ideal electrolytes were proposed. Then the detailed cation–anion, cation–solvent, and solvent–solvent interactions were presented. The resultant solvation structure from these interactions was depicted using physical–chemical descriptors. Finally, we established a correlation between solvation structure and electrochemical performance and concluded effective strategies for modulating solvation behavior. Several experimental demonstrations were discussed to show how adjusting the solvation structure can optimize battery performance.

The modulation of electrolytes to control solvation behavior has been at the forefront of electrolyte engineering in recent years. Even small electrolyte component interactions can lead to totally different electrochemical performance. In this regard, electrolyte modification can be an effective strategy to optimize battery performance. However, there are still some long‐standing challenges that preclude the development of solvation theory. First, inadequate characterization methods. Currently, we lack appropriate means to investigate the solvation behavior directly. The traditional NMR, Raman, and FTIR spectra characterization can only get the electrolyte bulk phase information; in fact, the localized electrolyte properties, such as localized coordination environment and interface ion arrangement, are difficult to decipher. Second, lack of theoretical models. It is undeniable that the current solvation and interface models are too simple. For instance, the solvation structure discussed above does not involve the solvation effect of anions. Also, only the first solvation shell is considered which ignores the second and higher order solvation shells. Obviously, the situation in the actual electrolyte is much more complex than the first shell. In addition, the existing interface model ignores several important variables such as the electric double layer at the interface and concentration gradients.

Therefore, we proposed some promising solutions to address the current tough missions of electrolyte solvation theory. 1) Multiscale characterization methods. To the best of our knowledge, both the solvation process and de‐solvation process are dynamically evolving. Combining the timescale, the dynamic de‐solvation process is possible to be parsed. For example, the ultrafast electron diffraction technology and ultrafast time‐resolved X‐ray diffraction technology, have not been utilized in the realm of battery thus far. In addition, combining the space scale, the detailed interface behavior of cations can be really seen. For instance, the cryo‐transmission electron microscope technology could achieve the capturing of transient interface information without damaging the electrode electrolyte interface. Therefore, combing the time and space scale characterizations, as well as the spectrum analysis, would benefit to deeply understand the solvation theory. 2) Machine learning and artificial intelligence. The cutting‐edge computer technology, such as the machine learning and artificial intelligence, allows us to simulate the solvation behavior of thousands of molecules in the electrolyte, thereby analyzing tendency and summarizing rules. Moreover, the machine learning technology is likely to screen the optimized anions, solvents, and additives to design the most compatible electrolyte system.

All in all, conducting electrolyte engineering by designing the solvation structure, is gradually attracting more and more research interests. Not only SIBs, other alkali metal ion batteries and aqueous ion batteries are also suitable for using solvation theory to adjust the electrolyte to optimize battery performance. Solvation theory and SEI theory complement each other, and gradually become the two carriages of electrolyte engineering to bridge the macro‐electrochemical performance and micro‐molecular dynamics. Compared to the SEI theory, the solvation structure design principle can be useful in electrolyte design, but more experimental verification of these principles is needed. We believe that combing the solvation theory and SEI theory opens a promising avenue for design compatible electrolyte in SIBs and other ion battery systems.

## Conflict of Interest

The authors declare no conflict of interest.

## References

[1] C. Delmas , Adv. Energy Mater. 2018, 8, 1703137.

[2] M. D. Slater , D. Kim , E. Lee , C. S. Johnson , Adv. Funct. Mater. 2013, 23, 947.

[3] K. M. Abraham , ACS Energy Lett. 2020, 5, 3544.

[4] N. Yabuuchi , K. Kubota , M. Dahbi , S. Komaba , Chem. Rev. 2014, 114, 11636. 25390643 10.1021/cr500192f

[5] K. West , B. Zachau‐christiansen , T. Jacobsen , S. Skaarup , J. Power Sources 1989, 26, 341.

[6] T. Liu , Y. Zhang , Z. Jiang , X. Zeng , J. Ji , Z. Li , X. Gao , M. Sun , Z. Lin , M. Ling , J. Zheng , C. Liang , Energy Environ. Sci. 2019, 12, 1512.

[7] A. Ponrouch , M. R. Palacin , Philos. Trans. R. Soc., A 2019, 377, 20180297. 10.1098/rsta.2018.0297 PMC6635625 31280715

[8] T. Perveen , M. Siddiq , N. Shahzad , R. Ihsan , A. Ahmad , M. I. Shahzad , Renewable Sustainable Energy Rev. 2020, 119, 109549.

[9] J. M. Lee , G. Singh , W. Cha , S. Kim , J. Yi , S.‐J. Hwang , A. Vinu , ACS Energy Lett. 2020, 5, 1939.

[10] G. Chang , Y. Zhao , L. Dong , D. P. Wilkinson , L. Zhang , Q. Shao , W. Yan , X. Sun , J. Zhang , J. Mater. Chem. A 2020, 8, 4996.

[11] M.‐S. Balogun , Y. Luo , W. Qiu , P. Liu , Y. Tong , Carbon 2016, 98, 162.

[12] D. A. Stevensa , J. R. Dahn , J. Electrochem. Soc. 2000, 147, 1271.

[13] Y. Niu , Y. Zhang , M. Xu , J. Mater. Chem. A 2019, 7, 15006.

[14] M. H. Han , E. Gonzalo , G. Singh , T. Rojo , Energy Environ. Sci. 2015, 8, 81.

[15] A. Ponrouch , D. Monti , A. Boschin , B. Steen , P. Johansson , M. R. Palacín , J. Mater. Chem. A 2015, 3, 22.

[16] H. Che , S. Chen , Y. Xie , H. Wang , K. Amine , X.‐Z. Liao , Z.‐F. Ma , Energy Environ. Sci. 2017, 10, 1075.

[17] G. Åvall , J. Mindemark , D. Brandell , P. Johansson , Adv. Energy Mater. 2018, 8, 1703036.

[18] S. Komaba , G. G. Eshetu , G. A. Elia , M. Armand , M. Forsyth , S. Komaba , T. Rojo , S. Passerini , Adv. Energy Mater. 2020, 10, 200093.

[19] S. Komaba , T. Ishikawa , N. Yabuuchi , W. Murata , A. Ito , Y. Ohsawa , ACS Appl. Mater. Interfaces 2011, 3, 4165. 22026720 10.1021/am200973k

[20] Z. Zeng , X. Jiang , R. Li , D. Yuan , X. Ai , H. Yang , Y. Cao , Adv. Sci. 2016, 3, 1600066. 10.1002/advs.201600066 PMC5039966 27711263

[21] Z. W. Zhang , Y. Li , R. Xu , W. Zhou , Y. Li , S. T. Oyakhire , Y. Wu , J. Xu , H. Wang , Z. Yu , D. T. Boyle , W. Huang , Y. Ye , H. Chen , J. Wan , Z. Bao , W. Chiu , Y. Cui , Science 2022, 75, 66. 10.1126/science.abi8703 34990230

[22] Z. Shadike , H. Lee , O. Borodin , X. Cao , X. Fan , X. Wang , R. Lin , S. M. Bak , S. Ghose , K. Xu , C. Wang , J. Liu , J. Xiao , X. Q. Yang , E. Hu , Nat. Nanotechnol. 2021, 16, 549. 33510453 10.1038/s41565-020-00845-5

[23] E. Peled , J. Electrochem. Soc. 1979, 126, 2047.

[24] J. Ming , Z. Cao , W. Wahyudi , M. Li , P. Kumar , Y. Wu , J.‐Y. Hwang , M. N. Hedhili , L. Cavallo , Y.‐K. Sun , L.‐J. Li , ACS Energy Lett. 2018, 3, 335.

[25] L. Zhou , Z. Cao , W. Wahyudi , J. Zhang , J.‐Y. Hwang , Y. Cheng , L. Wang , L. Cavallo , T. Anthopoulos , Y.‐K. Sun , H. N. Alshareef , J. Ming , ACS Energy Lett. 2020, 5, 766.

[26] L. Zhou , Z. Cao , J. Zhang , H. Cheng , G. Liu , G. T. Park , L. Cavallo , L. Wang , H. N. Alshareef , Y. K. Sun , J. Ming , Adv. Mater. 2021, 33, 2005993. 10.1002/adma.202005993 33470482

[27] Q. Li , Z. Cao , W. Wahyudi , G. Liu , G.‐T. Park , L. Cavallo , T. D. Anthopoulos , L. Wang , Y.‐K. Sun , H. N. Alshareef , J. Ming , ACS Energy Lett. 2020, 6, 69.

[28] S. Miertus , E. Scrocco , J. Tomasi , Chem. Phys. 1981, 55, 117.

[29] G. Kamath , R. W. Cutler , S. A. Deshmukh , M. Shakourian‐Fard , R. Parrish , J. Huether , D. P. Butt , H. Xiong , S. K. R. S. Sankaranarayanan , J. Phys. Chem. C 2014, 118, 13406.

[30] M. Shakourian‐Fard , G. Kamath , K. Smith , H. Xiong , S. K. R. S. Sankaranarayanan , J. Phys. Chem. C 2015, 119, 22747.

[31] T. A. Pham , K. E. Kweon , A. Samanta , V. Lordi , J. E. Pask , J. Phys. Chem. C 2017, 121, 21913.

[32] Y. Yamada , J. Wang , S. Ko , E. Watanabe , A. Yamada , Nat. Energy 2019, 4, 269.

[33] E. Wang , Y. Niu , Y.‐X. Yin , Y.‐G. Guo , ACS Mater. Lett. 2020, 3, 18.

[34] Y.‐S. Hu , Y. Lu , ACS Energy Lett. 2020, 5, 3633.

[35] J. Lee , Y. Lee , J. Lee , S. M. Lee , J. H. Choi , H. Kim , M. S. Kwon , K. Kang , K. T. Lee , N. S. Choi , ACS Appl. Mater. Interfaces 2017, 9, 3723. 28067499 10.1021/acsami.6b14878

[36] L. Zhou , Z. Cao , J. Zhang , Q. Sun , Y. Wu , W. Wahyudi , J. Y. Hwang , L. Wang , L. Cavallo , Y. K. Sun , H. N. Alshareef , J. Ming , Nano Lett. 2020, 20, 3247. 32319776 10.1021/acs.nanolett.9b05355

[37] Y. Wu , L. Xie , H. Ming , Y. Guo , J.‐Y. Hwang , W. Wang , X. He , L. Wang , H. N. Alshareef , Y.‐K. Sun , J. Ming , ACS Energy Lett. 2020, 5, 807.

[38] J. Ming , Z. Cao , Y. Wu , W. Wahyudi , W. Wang , X. Guo , L. Cavallo , J.‐Y. Hwang , A. Shamim , L.‐J. Li , Y.‐K. Sun , H. N. Alshareef , ACS Energy Lett. 2019, 4, 2613.

[39] Y. Zou , Z. Cao , J. Zhang , W. Wahyudi , Y. Wu , G. Liu , Q. Li , H. Cheng , D. Zhang , G. T. Park , L. Cavallo , T. D. Anthopoulos , L. Wang , Y. K. Sun , J. Ming , Adv. Mater. 2021, 33, 2102964. 10.1002/adma.202102964 34510582

[40] T. Zhang , Y. Li , N. Chen , Z. Wen , Y. Shang , Y. Zhao , M. Yan , M. Guan , F. Wu , R. J. A. A. M. Chen , Interfaces 2021, 13, 681. 10.1021/acsami.0c19075 33398985

[41] R. Younesi , G. M. Veith , P. Johansson , K. Edström , T. Vegge , Energy Environ. Sci. 2015, 8, 1905.

[42] C. A. Reed , Acc. Chem. Res. 1998, 31, 133.

[43] I. Krossing , I. Raabe , Angew. Chem., Int. Ed. 2004, 43, 2066. 10.1002/anie.200300620 15083452

[44] G. G. Eshetu , S. Grugeon , H. Kim , S. Jeong , L. Wu , G. Gachot , S. Laruelle , M. Armand , S. Passerini , ChemSusChem 2016, 9, 462. 26834069 10.1002/cssc.201501605

[45] Y. Sun , P. Shi , H. Xiang , X. Liang , Y. J. S. Yu , Small 2019, 15, 1805479. 10.1002/smll.201805479 30730107

[46] G. G. Eshetu , T. Diemant , M. Hekmatfar , S. Grugeon , R. J. Behm , S. Laruelle , M. Armand , S. Passerini , Nano Energy 2019, 55, 327.

[47] G. G. Eshetu , S. Grugeon , H. Kim , S. Jeong , L. Wu , G. Gachot , S. Laruelle , M. Armand , S. Passerini , ChemSusChem 2016, 9, 462. 26834069 10.1002/cssc.201501605

[48] D. Monti , E. Jonsson , A. Boschin , M. R. Palacin , A. Ponrouch , P. Johansson , Phys. Chem. Chem. Phys. 2020, 22, 22768. 33021285 10.1039/d0cp03639k

[49] S. An , M. W. Lee , N. Y. Kim , C. Lee , S. S. Al‐Deyab , S. C. James , S. S. Yoon , Appl. Phys. Lett. 2014, 105, 214102.

[50] P. Peljo , H. H. Girault , Energy Environ. Sci. 2018, 11, 2306. 10.1039/d4ee04560b PMC11753199 39866363

[51] A. L. Michan , M. Leskes , C. P. Grey , Chem. Mater. 2015, 28, 385.

[52] S. Solchenbach , G. Hong , A. T. S. Freiberg , R. Jung , H. A. Gasteiger , J. Electrochem. Soc. 2018, 165, A3304.

[53] C. Bommier , X. Ji , Small 2018, 14, 1703576. 10.1002/smll.201703576 29356418

[54] Z. Lin , Q. Xia , W. Wang , W. Li , S. Chou , InfoMat 2019, 1, 376.

[55] J. Sangster , J. Phase Equilib. Diffus. 2007, 28, 571.

[56] K. Nobuhara , H. Nakayama , M. Nose , S. Nakanishi , H. Iba , J. Power Sources 2013, 243, 585.

[57] B. Jache , P. Adelhelm , Angew. Chem., Int. Ed. 2014, 53, 10169. 10.1002/anie.201403734 25056756

[58] K. Li , J. Zhang , D. Lin , D. W. Wang , B. Li , W. Lv , S. Sun , Y. B. He , F. Kang , Q. H. Yang , L. Zhou , T. Y. Zhang , Nat. Commun. 2019, 10, 725. 30760713 10.1038/s41467-019-08506-5 PMC6374418

[59] H. Che , X. Yang , H. Wang , X.‐Z. Liao , S. S. Zhang , C. Wang , Z.‐F. Ma , J. Power Sources 2018, 407, 173.

[60] W. Zhang , L. Xing , J. Chen , H. Zhou , S. Liang , W. Huang , W. Li , J. Alloys Compd. 2020, 822, 153530.

[61] H. Wang , C. Wang , E. Matios , W. Li , Angew. Chem., Int. Ed. 2018, 57, 7734. 10.1002/anie.201801818 29693763

[62] X. Song , T. Meng , Y. Deng , A. Gao , J. Nan , D. Shu , F. Yi , Electrochim. Acta 2018, 281, 370.

[63] W. Wahyudi , V. Ladelta , L. Tsetseris , M. M. Alsabban , X. Guo , E. Yengel , H. Faber , B. Adilbekova , A. Seitkhan , A. H. Emwas , M. N. Hedhili , L. J. Li , V. Tung , N. Hadjichristidis , T. D. Anthopoulos , J. Ming , Adv. Funct. Mater. 2021, 31, 2101593.

[64] L. Chen , B. Kishore , M. Walker , C. E. J. Dancer , E. Kendrick , Chem. Commun. 2020, 56, 11609. 10.1039/d0cc03976d 32869777

[65] J. Feng , L. Ci , S. Xiong , RSC Adv. 2015, 5, 96649.

[66] Y. Yu , H. Che , X. Yang , Y. Deng , L. Li , Z.‐F. Ma , Electrochem. Commun. 2020, 110, 106635.

[67] J. Feng , Y. An , L. Ci , S. Xiong , J. Mater. Chem. A 2015, 3, 14539.

[68] S. S. Zhang , J. Power Sources 2006, 162, 1379.

[69] J. Y. Jang , Y. Lee , Y. Kim , J. Lee , S.‐M. Lee , K. T. Lee , N.‐S. Choi , J. Mater. Chem. A 2015, 3, 8332.

[70] H. Che , J. Liu , H. Wang , X. Wang , S. S. Zhang , X.‐Z. Liao , Z.‐F. Ma , Electrochem. Commun. 2017, 83, 20.

[71] S. A. Ferdousi , M. Hilder , A. Basile , H. Zhu , L. A. O'Dell , D. Saurel , T. Rojo , M. Armand , M. Forsyth , P. C. Howlett , ChemSusChem 2019, 12, 1700. 30740908 10.1002/cssc.201802988

[72] W. Fang , R. Jiang , H. Zheng , Y. Zheng , Y. Sun , X. Liang , H.‐F. Xiang , Y.‐Z. Feng , Y. Yu , Rare Met. 2020, 40, 433.

[73] C.‐H. Jo , J. U. Choi , H. Yashiro , S.‐T. Myung , J. Mater. Chem. A 2019, 7, 3903.

[74] M. Benchakar , R. Naéjus , C. Damas , J. Santos‐Peña , Electrochim. Acta 2020, 330, 135193.

[75] C. Cometto , G. Yan , S. Mariyappan , J.‐M. Tarascon , J. Electrochem. Soc. 2019, 166, A3723.

[76] K. W. Schroder , A. G. Dylla , L. D. Bishop , E. R. Kamilar , J. Saunders , L. J. Webb , K. J. Stevenson , J. Phys. Chem. Lett. 2015, 6, 2888. 26267175 10.1021/acs.jpclett.5b01216

[77] N. Sahai , D. A. Sverjensky , Geochim. Cosmochim. Acta 1997, 61, 2827. 10.1016/s0016-7037(97)00009-4 11541435

[78] X. Chen , Q. Zhang , Acc. Chem. Res. 2020, 53, 1992. 32883067 10.1021/acs.accounts.0c00412

[79] X. Chen , X.‐Q. Zhang , H.‐R. Li , Q. Zhang , Batteries Supercaps 2019, 2, 128.

[80] T. R. Griffiths , D. C. Pugh , Coord. Chem. Rev. 1979, 29, 129.

[81] M. Okoshi , Y. Yamada , A. Yamada , H. Nakai , J. Electrochem. Soc. 2013, 160, A2160.

[82] A. J. Smith , J. C. Burns , X. Zhao , D. Xiong , J. R. Dahn , J. Electrochem. Soc. 2011, 158, S23.

[83] X.‐Q. Zhang , X. Chen , L.‐P. Hou , B.‐Q. Li , X.‐B. Cheng , J.‐Q. Huang , Q. Zhang , ACS Energy Lett. 2019, 4, 411.

[84] W. Deng , W. Dai , X. Zhou , Q. Han , W. Fang , N. Dong , B. He , Z. J. A. E. L. Liu , ACS Energy Lett. 2020, 6, 115.

[85] J. Holoubek , M. Yu , S. Yu , M. Li , Z. Wu , D. Xia , P. Bhaladhare , M. S. Gonzalez , T. A. Pascal , P. J. A. E. L. Liu , ACS Energy Lett. 2020, 5, 1438.

[86] H. Yang , X. Chen , N. Yao , N. Piao , Z. Wang , K. He , H.‐M. Cheng , F. J. A. E. L. Li , ACS Energy Lett. 2021, 6, 1413.

[87] Z. Chen , Y. Tang , X. Du , B. Chen , G. Lu , X. Han , Y. Zhang , W. Yang , P. Han , J. Zhao , G. Cui , Angew. Chem., Int. Ed. 2020, 59, 21769. 10.1002/anie.202010423 32812326

[88] I. M. Riddlestone , A. Kraft , J. Schaefer , I. Krossing , Angew. Chem., Int. Ed. 2018, 57, 13982. 10.1002/anie.201710782 29266644

[89] J. Reiter , S. Jeremias , E. Paillard , M. Winter , S. Passerini , Phys. Chem. Chem. Phys. 2013, 15, 2565. 23302957 10.1039/c2cp43066e

[90] K. E. Gunderson‐Briggs , T. Ruther , A. S. Best , M. Kar , C. Forsyth , E. I. Izgorodiana , D. R. MacFarlane , A. F. Hollenkamp , Angew. Chem., Int. Ed. 2019, 58, 4390. 10.1002/anie.201813091 30632254

[91] A. Plewa‐Marczewska , T. Trzeciak , A. Bitner , L. Niedzicki , M. Dranka , G. Z. Żukowska , M. Marcinek , W. Wieczorek , Chem. Mater. 2014, 26, 4908.

[92] W. Geiger , F. Barrtere , Acc. Chem. Res. 2010, 43, 1030. 20345126 10.1021/ar1000023

[93] P. K. Pal , S. Chowdhury , M. G. B. Drew , D. Datta , New J. Chem. 2002, 26, 367.

[94] Y. Qin , Z. Chen , H. S. Lee , X. Q. Yang , K. Amine , J. Phys. Chem. C 2010, 114, 15202.

[95] J. Zheng , J. Xiao , M. Gu , P. Zuo , C. Wang , J.‐G. Zhang , J. Power Sources 2014, 250, 313.

[96] R. Parida , G. N. Reddy , A. Chakraborty , S. Giri , M. Jana , J. Chem. Inf. Model 2019, 59, 2159. 30794403 10.1021/acs.jcim.9b00035

[97] Y. Ma , Y. Zhou , C. Du , P. Zuo , X. Cheng , L. Han , D. Nordlund , Y. Gao , G. Yin , H. L. Xin , M. M. Doeff , F. Lin , G. Chen , Chem. Mater. 2017, 29, 2141.

[98] H. S. Lee , Z. F. Ma , X. Q. Yang , X. Sun , J. McBreen , J. Electrochem. Soc. 2004, 151, A1429.

[99] Y. Ma , Z. Zhou , C. Li , L. Wang , Y. Wang , X. Cheng , P. Zuo , C. Du , H. Huo , Y. Gao , G. Yin , Energy Storage Mater. 2018, 11, 197.

[100] L. Cao , D. Li , E. Hu , J. Xu , T. Deng , L. Ma , Y. Wang , X. Q. Yang , C. Wang , J. Am. Chem. Soc. 2020, 142, 21404. 33290658 10.1021/jacs.0c09794

[101] W. R. McKinnon , J. R. Dahn , J. Electrochem. Soc. 1985, 132, 364.

[102] S. K. Jeong , M. Inaba , Y. Iriyama , T. Abe , Z. Ogumi , Electrochem. Solid‐State Lett. 2003, 6, A13.

[103] S. Terada , T. Mandai , R. Nozawa , K. Yoshida , K. Ueno , S. Tsuzuki , K. Dokko , M. Watanabe , Phys. Chem. Chem. Phys. 2014, 16, 11737. 24810659 10.1039/c4cp00746h

[104] C. Guo , K. Zhang , Q. Zhao , L. Pei , J. Chen , Chem. Commun. 2015, 51, 10244. 10.1039/c5cc02251g 26022356

[105] R. Cao , K. Mishra , X. Li , J. Qian , M. H. Engelhard , M. E. Bowden , K. S. Han , K. T. Mueller , W. A. Henderson , J.‐G. Zhang , Nano Energy 2016, 30, 825.

[106] L. Schafzahl , I. Hanzu , M. Wilkening , S. A. Freunberger , ChemSusChem 2017, 10, 401. 27860417 10.1002/cssc.201601222

[107] K. Takada , Y. Yamada , E. Watanabe , J. Wang , K. Sodeyama , Y. Tateyama , K. Hirata , T. Kawase , A. Yamada , ACS Appl. Mater. Interfaces 2017, 9, 33802. 28766928 10.1021/acsami.7b08414

[108] M. He , K. C. Lau , X. Ren , N. Xiao , W. D. McCulloch , L. A. Curtiss , Y. Wu , Angew. Chem., Int. Ed. 2016, 55,15310. 10.1002/anie.201608607 27809386

[109] J. Wang , Y. Yamada , K. Sodeyama , E. Watanabe , K. Takada , Y. Tateyama , A. Yamada , Nat. Energy 2017, 3, 22.

[110] M. Li , C. Wang , Z. Chen , K. Xu , J. Lu , Chem. Rev. 2020, 120, 6783. 32022546 10.1021/acs.chemrev.9b00531

[111] Y. Li , Y. Yang , Y. Lu , Q. Zhou , X. Qi , Q. Meng , X. Rong , L. Chen , Y.‐S. Hu , ACS Energy Lett. 2020, 5, 1156.

[112] J. Zheng , S. Chen , W. Zhao , J. Song , M. H. Engelhard , J.‐G. Zhang , ACS Energy Lett. 2018, 3, 315.

[113] Y. Jin , Y. Xu , P. M. L. Le , T. D. Vo , Q. Zhou , X. Qi , M. H. Engelhard , B. E. Matthews , H. Jia , Z. Nie , C. Niu , C. Wang , Y. Hu , H. Pan , J.‐G. Zhang , ACS Energy Lett. 2020, 5, 3212.

[114] Y. Yamada , K. Furukawa , K. Sodeyama , K. Kikuchi , M. Yaegashi , Y. Tateyama , A. Yamada , J. Am. Chem. Soc. 2014, 136, 5039. 24654781 10.1021/ja412807w

[115] C. Yang , J. Chen , X. Ji , T. P. Pollard , X. Lu , C. J. Sun , S. Hou , Q. Liu , C. Liu , T. Qing , Y. Wang , O. Borodin , Y. Ren , K. Xu , C. Wang , Nature 2019, 569, 245. 31068723 10.1038/s41586-019-1175-6

[116] L. Jiang , Y. Lu , C. Zhao , L. Liu , J. Zhang , Q. Zhang , X. Shen , J. Zhao , X. Yu , H. Li , X. Huang , L. Chen , Y.‐S. Hu , Nat. Energy 2019, 4, 495.

[117] L. Jiang , L. Liu , J. Yue , Q. Zhang , A. Zhou , O. Borodin , L. Suo , H. Li , L. Chen , K. Xu , Y. S. Hu , Adv. Mater. 2020, 32, 1904427. 10.1002/adma.201904427 31782981

[118] Z. Zhao , F. Li , J. Zhao , G. Ding , J. Wang , X. Du , Q. Zhou , G. Hou , G. Cui , Adv. Funct. Mater. 2020, 30, 2000347.

[119] W. L. Jorgensen , D. S. Maxwell , J. J. J. o. t. A. C. S. Tirado‐Rives , J. Am. Chem. Soc. 1996, 118, 11225.

[120] S. Pronk , S. Páll , R. Schulz , P. Larsson , P. Bjelkmar , R. Apostolov , M. R. Shirts , J. C. Smith , P. M. Kasson , D. J. B. Van Der Spoel , Bioinformatics 2013, 29, 845. 23407358 10.1093/bioinformatics/btt055 PMC3605599

[121] H. J. Berendsen , J. v. Postma , W. F. van Gunsteren , A. DiNola , J. R. J. T. J. o. c. p. Haak , J. Chem. Phys. 1984, 81, 3684.

[122] G. Bussi , D. Donadio , M. J. T. J. o. c. p. Parrinello , J. Phys. Chem. 2007, 126, 014101. 10.1063/1.2408420 17212484

[123] U. Essmann , L. Perera , M. L. Berkowitz , T. Darden , H. Lee , L. Pedersen , J. Phys. Chem. 1995, 103, 8577.

[124] W. Humphrey , A. Dalke , K. Schulten , J. Mol. Graphics 1996, 14, 33. 10.1016/0263-7855(96)00018-5 8744570

[125] A. Poater , B. Cosenza , A. Correa , S. Giudice , F. Ragone , V. Scarano , L. Cavallo , Eur. J. Inorganic Chem. 2009, 2009, 1759.

[126] S. Wu , B. Su , M. Sun , S. Gu , Z. Lu , K. Zhang , D. Y. W. Yu , B. Huang , P. Wang , C. S. Lee , W. Zhang , Adv. Mater. 2021, 33, 2102390. 10.1002/adma.202102390 34463369

[127] Z. Tian , V. S. Kale , Y. Wang , S. Kandambeth , J. Czaban‐Jozwiak , O. Shekhah , M. Eddaoudi , H. N. Alshareef , J. Am. Chem. Soc. 2021, 143, 19178. 34739750 10.1021/jacs.1c09290

[128] J. Rouxel , Mater. Sci. Eng. 1977, 31, 277.

[129] A. Mendibour , C. Delmas , P. Hagenmuller , J. Solid‐State Chem. 1985, 57, 323.

[130] J. B. Goodenough , H. Y.‐P. Hong , J. A. Kafalas , Mater. Res. Bull. 1976, 11, 203.

[131] F. Bragg , C. Gottfried , J. West , Z. Krist. 1931, 77, 255.

[132] S. H. Strauss , Chem. Rev. 1993, 93, 927.

[133] S. A. Hashmi , S. Chandra , Mater. Sci. Eng. 1995, B34, 18.

[134] T. B. Kim , C. W. Parka , H. S. Ryu , H. J. Ahn , Mater. Sci. Forum 2005, 486, 638.

[135] Z. Jian , W. Han , X. Lu , H. Yang , Y. S. Hu , J. Zhou , Z. Zhou , J. Li , W. Chen , D. Chen , L. Q. Chen , Adv. Energy Mater. 2013, 3, 156.

[136] R. Alcántara , P. Lavela , G. F. Ortiz , J. L. Tirado , Electrochem. Solid‐State Lett. 2005, 8, A222.

[137] A. Ponrouch , E. Marchante , M. Courty , J. M. Tarascon , M. R. Palacín , Energy Environ. Sci. 2012, 5, 8572.

[138] D. Monti , E. Jónsson , M. R. Palacín , P. Johansson , J. Power Sources 2014, 245, 630.

[139] X. Chen , X. Shen , T. Z. Hou , R. Zhang , H. J. Peng , Q. Zhang , Chem 2020, 6, 2242.

